# Identification of a Lipoteichoic Acid Glycosyltransferase Enzyme Reveals that GW-Domain-Containing Proteins Can Be Retained in the Cell Wall of Listeria monocytogenes in the Absence of Lipoteichoic Acid or Its Modifications

**DOI:** 10.1128/JB.00116-16

**Published:** 2016-07-13

**Authors:** Matthew G. Percy, Eleni Karinou, Alexander J. Webb, Angelika Gründling

**Affiliations:** Section of Microbiology and MRC Centre for Molecular Bacteriology and Infection, Imperial College London, London, United Kingdom; The University of Chicago

## Abstract

Listeria monocytogenes is a foodborne Gram-positive bacterial pathogen, and many of its virulence factors are either secreted proteins or proteins covalently or noncovalently attached to the cell wall. Previous work has indicated that noncovalently attached proteins with GW (glycine-tryptophan) domains are retained in the cell wall by binding to the cell wall polymer lipoteichoic acid (LTA). LTA is a glycerol phosphate polymer, which is modified in L. monocytogenes with galactose and d-alanine residues. We identified Lmo0933 as the cytoplasmic glycosyltransferase required for the LTA glycosylation process and renamed the protein GtlA, for glycosyltransferase LTA A. Using L. monocytogenes mutants lacking galactose or d-alanine modifications or the complete LTA polymer, we show that GW domain proteins are retained within the cell wall, indicating that other cell wall polymers are involved in the retention of GW domain proteins. Further experiments revealed peptidoglycan as the binding receptor as a purified GW domain fusion protein can bind to L. monocytogenes cells lacking wall teichoic acid (WTA) as well as purified peptidoglycan derived from a wild-type or WTA-negative strain. With this, we not only identify the first enzyme involved in the LTA glycosylation process, but we also provide new insight into the binding mechanism of noncovalently attached cell wall proteins.

**IMPORTANCE** Over the past 20 years, a large number of bacterial genome sequences have become available. Computational approaches are used for the genome annotation and identification of genes and encoded proteins. However, the function of many proteins is still unknown and often cannot be predicted bioinformatically. Here, we show that the previously uncharacterized Listeria monocytogenes gene *lmo0933* likely codes for a glycosyltransferase required for the decoration of the cell wall polymer lipoteichoic acid (LTA) with galactose residues. Using L. monocytogenes mutants lacking LTA modifications or the complete polymer, we show that specific cell wall proteins, often associated with virulence, are retained within the cell wall, indicating that additional cell wall polymers are involved in their retention.

## INTRODUCTION

*L*isteria monocytogenes is a foodborne Gram-positive bacterial pathogen and the causative agent of human listeriosis, with immunocompromised individuals, pregnant women, and neonates being at particular risk of infection (1). As part of its life cycle, L. monocytogenes can enter phagocytic and nonphagocytic cells. Following this, the bacterium escapes from the vacuole, replicates within the host cell cytoplasm, and then spreads from cell to cell via actin-based motility (2, 3). Numerous L. monocytogenes proteins required for this intricate intracellular life cycle and for the pathogenesis of this organism have been characterized over the past decades, and many of these virulence factors are either secreted or cell wall-associated proteins (4–6).

Proteins that are retained within the cell wall of Gram-positive bacteria are either covalently bound to peptidoglycan through a reaction catalyzed by sortase enzymes or retained within the cell wall through a noncovalent interaction with peptidoglycan or other cell wall components. Noncovalently bound proteins contain specific cell wall binding domains, and in the case of L. monocytogenes, these are GW (glycine-tryptophan) modules or WXL or LysM domains (see reviews in references 6 and 7). While the function of the WXL-domain-containing proteins is unknown, most of the proteins containing GW or LysM domains have additional enzymatic domains predicted to contain peptidoglycan hydrolytic activity. An exception is the GW-domain-containing protein internalin B (InlB), which aids bacteria in the entry process of hepatocytic, endothelial, and epithelial cells (8–10). GW domains are around 80 amino acids in length, and the name is derived from the presence of a conserved glycine-tryptophan (GW) dipeptide within the binding domain (6, 7, 11). The GW domains are often repeated and not present as single domains. For instance, InlB contains three C-terminal GW domains and the autolysin Ami has eight C-terminal GW domains (11). In previous work, a correlation between the number of GW domains and the strength of interaction with the cell wall has been observed as InlB was found in both the cell wall and supernatant fraction, while the Ami protein, with a larger number of GW domains, was found exclusively in the cell wall fraction (11). The cell wall polymer lipoteichoic acid (LTA) has been implicated as the binding receptor for GW-domain-containing proteins (12). In addition, it has also been described that InlB binds to glycosaminoglycans present on host cell surfaces (13, 14), indicating that sugar moieties are recognized by the GW domains, and this was further investigated in the present study.

The cell wall of L. monocytogenes is typical for a Gram-positive bacterium belonging to the phylum Firmicutes. It is composed of a thick peptidoglycan layer and teichoic acids (TAs). TAs are further grouped into wall teichoic acid (WTA), a polymer covalently linked to the peptidoglycan layer, and lipoteichoic acid (LTA), a polymer anchored to the outer leaflet of the cytoplasmic membrane by a glycolipid anchor (15, 16). The chemical structure of WTA can vary between different Listeria serovars. It is either a polyribitol-phosphate (RboP) polymer substituted with *N*-acetylglucosamine (GlcNAc) and/or rhamnose (Rha) residues (17–19) or a polymer made up of RboP and GlcNAc repeating units, which can be further decorated with galactose and glucose residues (19, 20). In all cases, the WTA polymer is covalently linked via a conserved sugar-containing linker unit to the peptidoglycan layer. The WTA polymer itself is polymerized within the cytoplasm of the cell on an undecaprenyl-phosphate (C_55_-P) lipid carrier molecule, and its synthesis is initiated by the TagO enzyme and the production of a conserved linker unit. Two TagO enzymes with redundant functions have been identified in L. monocytogenes, and only depletion of both enzymes resulted in the abrogation of WTA production in the L. monocytogenes strain EGD-e (21). Less variation is seen in the chemical structure of the LTA polymer (22, 23). It is a polyglycerol phosphate polymer, also referred to as type I LTA, further decorated with d-alanine and galactose residues in L. monocytogenes strains (15, 22–24). Most enzymes required for LTA production have now been identified; the proteins encoded in the *lafA-lafB-lafC* (*lafA-C*) operon (LTA anchor formation) are required for the production of the glycolipid anchor (25). The glycerol phosphate backbone is polymerized on the outside of the cell, and the GroP subunits are derived from the head group of the phospholipid phosphatidylglycerol phosphate (26, 27). In L. monocytogenes, the LTA backbone is synthesized by a two-enzyme system, where the LTA primase LtaP initiates the production of the glycerol phosphate backbone by the addition of the first glycerol phosphate subunit to the glycolipid anchor and the LTA synthase LtaS extends the chain by the repeated addition of glycerol phosphate subunits (24, 25, 28). The proteins encoded in the *dltA-dltB-dltC-dltD* (*dltA-D*) operon are responsible for the addition of the d-alanine modifications on LTA (29); however, the enzymes responsible for the modification of LTA with galactose residues have not yet been identified.

**FIG 1:**
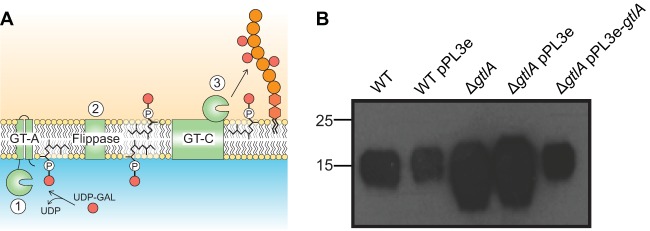
Model for the glycosylation process of type I LTA in L. monocytogenes and LTA production in the WT and a *gtlA* mutant strain. (A) Model for the glycosylation process of type I LTA. In this model, an enzyme with a cytoplasmically located glycosyltransferase (GT) domain, likely with a GT-A fold, utilizes UDP-Gal as the substrate to generate the C_55_-P-Gal lipid intermediate (step 1). Following this, the lipid-linked sugar intermediate is transferred across the membrane, presumably with the aid of a dedicated lipid flippase (step 2), where a GT with an extracellular catalytic domain and likely assuming a GT-C fold links the sugar onto LTA (step 3). It is of note that none of the enzymes has as of yet been identified in L. monocytogenes or any other bacterium producing type I LTA. This figure was adapted from Percy et al. (15). (B) LTA detection by Western blotting. Extracts from strains 10403S (WT), 10403S/pPL3e (WT pPL3e), 10403S Δ*gtlA* (Δ*gtlA*), 10403S Δ*gtlA*/pPL3e (Δ*gtlA* pPL3e), and 10403S Δ*gtlA*/pPL3e-*gtlA* (Δ*gtlA* pPL3e-*gtlA*) were prepared and separated in 15% PAA gels as described in Materials and Methods. The LTA polymer was detected by Western blotting using a polyglycerol phosphate-specific monoclonal antibody; positions of protein standards (in kilodaltons) are indicated on the left-hand side. Three independent experiments were performed, and a representative result is shown.

Based on biochemical studies performed in the 1980s, a mechanism for the incorporation of glycosyl residues into type I LTA has been proposed and also recently reviewed (15, 30–33) (Fig. 1A). In this model, a cytoplasmic glycosyltransferase (GT) utilizes a nucleotide-activated sugar to form a C_55_-P-sugar intermediate. Next, this intermediate is transported across the membrane by a flippase and in a final step a second GT with extracellular activity links the sugar onto LTA (32, 33) (Fig. 1A). The lipopolysaccharide (LPS) glycosylation process in Escherichia coli with 4-amino-4-deoxy-l-arabinose (l-Ara4N) and glycosylation processes of complex cell wall polymers in Mycobacterium tuberculosis proceed through similar mechanisms (34–36).

Here, we identified a glycosyltransferase involved in the LTA glycosylation process. Using L. monocytogenes mutant strains lacking either the d-alanine or glycosyl modifications or the complete LTA polymer, we show that LTA is not absolutely essential for the retention of the GW-repeat-domain-containing cell wall proteins InlB and Ami. We present experimental evidence that suggests that GW domain proteins are instead retained in the cell wall by binding to the peptidoglycan polymer. With this, we not only identify the first enzyme involved in the glycosylation process of type I LTA, but we also provide new insight into the binding mechanism of noncovalently attached cell wall proteins.

## MATERIALS AND METHODS

### Bacterial strains and growth conditions.

All strains and plasmids used in this study are listed along with the corresponding references in Table S1 in the supplemental material. Escherichia coli strains were grown in Luria-Bertani (LB) medium and Listeria monocytogenes strains in brain heart infusion (BHI) medium at 37°C unless otherwise specified. Where appropriate, the medium was supplemented with the following antibiotics: for E. coli cultures, ampicillin (Amp) at 100 μg/ml, kanamycin (Kan) at 30 μg/ml, tetracycline (Tet) at 10 μg/ml, and chloramphenicol (Cam) at 20 μg/ml; for L. monocytogenes cultures, chloramphenicol (Cam) at 7.5 to 10 μg/ml, erythromycin (Erm) at 5 μg/ml, and streptomycin (Strep) at 200 μg/ml for conjugation experiments.

### Strain and plasmid construction.

All primers used in this study are listed in Table S2 in the supplemental material. Allelic exchange vectors pKSV7Δ*dltA*, pKSV7Δ*tagO1*, pKSV7Δ*tagO2*, and pKSV7Δ*gtlA* (*lmo0933*) were constructed for the production of markerless in-frame deletions in L. monocytogenes genes *dltA*, *tagO1*, *tagO2*, and *gtlA*, respectively. For all constructs, an approximately 1-kb DNA region upstream of the gene and the first 30 bases of the gene were fused by splicing overlap extension (SOE) PCR (37) to the last 30 bases of the gene and an approximately 1-kb DNA fragment downstream of the gene. Chromosomal DNA isolated from L. monocytogenes strain 10403S was used as the DNA template in all PCRs. For construction of plasmid pKSV7Δ*dltA*, primer pairs 932/933 and 934/935 were used for the first round of PCRs and primer pair 932/935 for the second round. The PCR product was subsequently digested with enzymes KpnI and BamHI and ligated with vector pKSV7, which had been cut with the same enzymes. Plasmids pKSV7Δ*tagO1*, pKSV7Δ*tagO2*, and pKSV7Δ*gtlA* were constructed using a similar method but with primers 530/531/532/533 (Δ*tagO1*), 537/538/539/540 (Δ*tagO2*), and 1298/1299/1300/1301 (Δ*gtlA*). This created the allelic exchange vectors pKSV7Δ*dltA*, pKSV7Δ*tagO1*, pKSV7Δ*tagO2*, and pKSV7Δ*gtlA*, which were initially recovered in E. coli strain XL1-Blue, yielding strains ANG1646, ANG1380, ANG1381, and ANG2222, respectively. These plasmids were then introduced by electroporation into L. monocytogenes strain 10403S, and the allelic exchange procedure was subsequently performed as described previously (38). Strains with the expected gene deletions were identified by PCR, and this resulted in the construction of strains 10403S Δ*dltA* (ANG1783), 10403S Δ*tagO1* (ANG1387), 10403S Δ*tagO2* (ANG1388), and 10403S Δ*gtlA* (ANG2325). For construction of strain 10403S Δ*tagO1-2* (ANG1784) with deletions of both *tagO* genes, plasmid pKSV7Δ*tagO1* was introduced into strain 10403S Δ*tagO2* and the allelic exchange procedure performed as described above. Strains 10403S Δ*tagO1-2* and 10403S Δ*ltaS* (constructed in a previous study [25]) have severe growth defects, and to improve the growth of these strains they were passaged several times at 37°C, yielding the suppressor strains 10403S Δ*tagO1-2*_sup_ (ANG2350) and 10403S Δ*ltaS*_sup_ (ANG2337). For construction of strain 10403S Δ*ltaP* Δ*ltaS*_sup_ (ANG3465), the allelic exchange plasmid pKSV7Δ*lmo0644* (Δ*ltaP*) (25) was electroporated into L. monocytogenes strain 10403S Δ*ltaS*_sup_ and the allelic exchange procedure performed as described above.

Plasmids pPL3e-*gtlA*, pPL3-*tagO1*, and pHPL3-*tagO2* were constructed for gene complementation analysis. For construction of plasmid pPL3e-*gtlA*, the *gtlA* gene and its upstream promoter region were amplified with primer pair 1421/1422; the resulting PCR product was digested with BamHI and KpnI and ligated with vector pPL3e, which had been cut with the same enzymes. The resulting plasmid, pPL3e-*gtlA*, was recovered in E. coli strain XL1-Blue (ANG2322) and subsequently introduced into strain 10403S Δ*gtlA* by electroporation, generating strain 10403S Δ*gtlA*/pPL3e-*gtlA* (ANG2495). The empty pPL3e vector was also introduced into wild-type (WT) strain 10403S and strain 10403S Δ*gtlA*, yielding control strains ANG2498 and ANG2496, respectively. For construction of plasmids pPL3-*tagO1* and pHPL3-*tagO2*, the *tagO1* gene, including its upstream promoter region, was amplified with primer pair 655/679 and the *tagO2* gene without the promoter region was amplified with primer pair 657/658. The resulting PCR products were digested with enzymes BamHI and KpnI and ligated with plasmids pPL3 and pHPL3 (containing the constitutive hyper spac promoter), respectively. The resulting plasmids, pPL3-*tagO1* and pHPL3-*tagO2*, were recovered in E. coli strain XL1-Blue, yielding strains ANG1394 and ANG1395. Plasmids pHPL3, pPL3-*tagO1*, and pHPL3-*tagO2* were then introduced into the E. coli strain SM10, yielding strains ANG1375, ANG1807, and ANG1808, respectively. Plasmids pHPL3, pPL3-*tagO1*, and pHPL3-*tagO2* were subsequently moved by conjugation into strain 10403S Δ*tagO1-2*, yielding strains ANG1911, ANG1832, and ANG1833, respectively.

Plasmid pHPL3-*inlB*-GW-His was constructed for the expression of the C-terminally His-tagged InlB protein in L. monocytogenes under the control of the constitutive hyper spac promoter. Plasmid pHPL3-*inlB*-GW_Ami_-His was constructed for the expression of the C-terminally His-tagged InlB variant in which its three native GW domains were replaced with the eight GW domains of the Ami protein. For construction of plasmid pHPL3-*inlB*-GW-His, the *inlB* gene was amplified from 10403S chromosomal DNA using primer pair 835/837 and the C-terminal His tag was introduced as part of the primer sequence. The PCR product was cut with enzymes BamHI and SalI and inserted into plasmid pHPL3, which had been cut with the same enzymes. The resulting plasmid, pHPL3-*inlB*-GW-His, was recovered in E. coli strain XL1-Blue, yielding strain ANG1551. SOE PCR was used to create the insert *inlB*-GW_Ami_-His for the construction of plasmid pHPL3-*inlB*-GW_Ami_-His. Specifically, the 5′ *inlB* fragment was amplified with primer pair 835/1172 and the sequence coding for the GW domains of the Ami protein with primer pair 1173/1119. The PCR products were fused in a second round of PCR using primer pair 835/1119. The resulting PCR product was cut with enzymes BamHI and SalI and inserted into plasmid pHPL3. The resulting plasmid, pHPL3-*inlB*-GW_Ami_-His, was initially recovered in E. coli strain XL1-Blue, yielding strain ANG1975. Plasmids pHPL3-*inlB*-GW-His and pHPL3-*inlB*-GW_Ami_-His were either introduced into the different Listeria strains by electroporation or first introduced into E. coli strain SM10, yielding strains ANG1625 and ANG2014, and subsequently introduced into the different Listeria strains by conjugation. Specifically plasmids pHPL3, pHPL3-*inlB*-GW-His, and pHPL3-*inlB*-GW_Ami_-His were introduced into the WT L. monocytogenes 10403S strain, yielding strains 10403S/pHPL3 (ANG1414), 10403S/pHPL3-*inlB*-GW-His (ANG1626), and 10403S/pHPL3-*inlB*-GW_Ami_-His (ANG2015). Plasmids pHPL3-*inlB*-GW-His and pHPL3-*inlB*-GW_Ami_-His were also introduced into L. monocytogenes strains 10403S Δ*gtlA*, 10403S Δ*dltA*, 10403S Δ*ltaS*_sup_, 10403S Δ*ltaP* Δ*ltaS*_sup_, and 10403S Δ*tagO1-2*_sup_, yielding strains 10403S Δ*gtlA*/pHPL3-*inlB*-GW-His (ANG3330) and 10403S Δ*gtlA*/pHPL3-*inlB*-GW_Ami_-His (ANG3331), 10403S Δ*dltA*/pHPL3-*inlB*-GW-His (ANG3333) and 10403S Δ*dltA*/pHPL3-*inlB*-GW_Ami_-His (ANG3334), 10403S Δ*ltaS*_sup_/pHPL3-*inlB*-GW-His (ANG1630) and 10403S Δ*ltaS*_sup_/pHPL3-*inlB*-GW_Ami_-His (ANG2037), 10403S Δ*ltaP* Δ*ltaS*_sup_/pHPL3-*inlB*-GW-His (ANG3325) and 10403S Δ*ltaP* Δ*ltaS*_sup_/pHPL3-*inlB*-GW_Ami_-His (ANG3326), and 10403S Δ*tagO1-2*_sup_/pHPL3-*inlB*-GW-His (ANG2513) and 10403S Δ*tagO1-2*_sup_/pHPL3-*inlB*-GW_Ami_-His (ANG2514), respectively.

Plasmid pVL847 is an E. coli vector used for the expression and purification of the N-terminally His-tagged maltose binding protein (His-MBP). Plasmid pVL847-GW_Ami_ was constructed for the expression and purification of the His-MBP–GW_Ami_ fusion protein, in which the eight GW domains of the Ami protein are fused to the C terminus of the His-MBP protein. Initially the sequence coding for the Ami GW domains was amplified with primer pair 1320/1321, and the resulting PCR product was cut with enzymes SacI and SalI and inserted in plasmid pQ30-GFP. The resulting plasmid, pQE30-GFP-GW_Ami_, was recovered in E. coli strain XL1-Blue, yielding strain ANG2157. Plasmid pQE30-GFP-GW_Ami_ and primer pair 1806/1807 were subsequently used in a PCR to amplify the GW_Ami_ region and the resulting product was cut with enzymes NdeI and BamHI and ligated with plasmid pVL847, which had been cut with the same enzymes. This generated plasmid pVL847-GW_Ami_, which was recovered in E. coli strain in XL1-Blue, generating strain ANG3179. For protein expression and purification, plasmids pVL847 and pVL847-GW_Ami_ were introduced into E. coli strain BL21(DE3), generating strains ANG2890 and ANG3181, respectively. The DNA sequences of all inserts were confirmed by automated fluorescence sequencing at the MRC Clinical Sciences Centre Genomics Core Laboratory, Imperial College London or at GATC Biotech, Ltd.

### LTA isolation and NMR analysis.

Cultures of L. monocytogenes strains 10403S, 10403S Δ*gtlA*, and 10403S Δ*gtlA*/pPL3e-*gtlA* were grown overnight at 37°C in 3 liters of BHI medium, and bacteria were pelleted by centrifugation. The LTA was extracted and purified by hydrophobic interaction chromatography using a 24- by 1.6-cm octyl Sepharose column as previously described (26). LTA-containing fractions were identified by Western blotting using a polyglycerol phosphate-specific antibody (26), combined, and extensively dialyzed against double-distilled water (ddH_2_O) at 4°C. The sample was then lyophilized and stored at −20°C. To analyze the LTA by one-dimensional (1D) ^1^H nuclear magnetic resonance (NMR), 2 mg of purified LTA was suspended in 500 μl D_2_O of 99.96% purity and lyophilized. The LTA was suspended and lyophilized once more in this way and finally suspended in 500 μl of D_2_O of 99.99% purity. NMR spectra were acquired on a 600-MHz Bruker Avance III spectrometer equipped with a TCI cryoprobe. For quantification, 1D spectra were recorded with a 5-s recycle delay and approximately 45° proton flip angle to ensure accurate integration. The data were analyzed in Topspin 3.1 (Bruker Biospin, Ltd.). For each strain, three independent LTA extractions were performed and analyzed by NMR, and spectra were annotated as previously reported (39–41). To determine the chain length and degree of backbone substitution, the area under each peak assigned to an LTA component was integrated. The peak at 4.3 ppm derived from the nonexchangeable proton associated with the CH group of d-Ala was used as a reference and set to 1. Next, the total integration values for peaks belonging to specific LTA components (GroP, d-Ala, Gal, or fatty acids) were calculated. Following this, a proton adjusted value was determined for LTA components by taking the number of nonexchangeable protons for each component into account—that is, 58 protons for the lipid anchor, 5 for GroP, 4 for d-Ala, and 7 for Gal. To determine the chain length of LTA, the proton-adjusted value for GroP was divided by the proton-adjusted value for the lipid anchor. To determine the percentage of substitution, the proton-adjusted value for d-Ala or Gal was divided by the proton-adjusted value for GroP and multiplied by 100. The average values and standard deviations from three independent experiments were calculated and plotted. Statistical significance was determined using a two-tailed unpaired Student *t* test.

### L. monocytogenes growth curves and motility assay.

Growth curves with *tagO* mutant strains were undertaken as previously described (25). Briefly, WT strain 10403S and the *tagO* mutant L. monocytogenes strains were grown overnight at 30°C in 4 ml BHI medium. The next day, cultures were diluted to a starting optical density at 600 nm (OD_600_) of 0.07 in 25 ml of BHI medium and incubated with shaking at 37°C, and OD_600_ values were determined at timed intervals. Growth curves were performed in duplicate, and a representative graph is shown. Growth curves with the *gtlA* mutant and complementation strain were performed in 96-well format in a SPECTROStar Nano 96-well plate reader (BMG Labtech). The indicated strains were grown overnight at 37°C in 5 ml BHI medium. The next day, cultures were diluted in triplicate 1:50 into 200 μl of fresh BHI and incubated at 37°C with shaking for 10 h, and OD_600_ readings were taken every 30 min. The background reading of wells containing BHI medium only was subtracted from all readings and the average readings from the triplicate samples plotted. The experiment was performed in duplicate, and a representative result is shown. For motility assays, the indicated L. monocytogenes strains were grown overnight in 5-ml BHI cultures. The next day, the culture was adjusted to an OD_600_ of 5, 2.5 μl was used to stab inoculate low-agar (0.3% agar) BHI plates, and the plates were subsequently incubated for 18 h at 30°C. Images were taken using a ChemiDoc Touch imaging system (Bio-Rad). Experiments were performed in triplicate, and a representative result is shown.

### Cell fractionation and Western blot analysis.

WT and mutant L. monocytogenes strains were grown overnight in 5 ml BHI medium at 30°C. Samples for the detection of cell-associated LTA were prepared and separated on 15% polyacrylamide (PAA) gels as previously described (25). The LTA was detected by Western blotting using a polyglycerol phosphate-specific monoclonal LTA antibody (clone 55 from Hycult Biotechnology) and a horseradish peroxidase (HRP)-conjugated anti-mouse antibody (Cell Signaling Technology) at 1:4,000 and 1:10,000 dilutions, respectively. To assess the cellular localization of reporter proteins, the different L. monocytogenes strains were grown overnight in 5 ml BHI medium at 37°C (or 30°C for the 10403S Δ*tagO1-2*_sup_/pHPL3-*inlB*-GW-His [ANG2513] and 10403S Δ*tagO1-2*_sup_/pHPL3-*inlB*_Ami_-His [ANG2514] strains). The next day, 1.2 ml of these cultures was centrifuged at 17,000 × *g* for 10 min to harvest the bacteria. Following this, 1 ml of the supernatant was transferred to a fresh tube (supernatant fraction). The remaining supernatant was aspirated, leaving a cell pellet (cell-associated fraction). To prepare the cell-associated protein fraction, the cell pellet was suspended in 2× protein sample buffer normalized for culture density; that is, the pellet from a 1.2-ml culture with an OD_600_ of 2 was suspended in 120 μl sample buffer. To prepare the supernatant fraction, 100 μl of cold trichloroacetic acid (TCA) was added to the 1-ml supernatant fraction, and the sample was vortexed and incubated on ice for 1 h. Then the samples were centrifuged at 17,000 × *g* for 10 min, and the pellet was washed twice with 1 ml cold acetone, followed by vortexing and incubation on ice for 1 h. The samples were centrifuged as before, the supernatant aspirated, and the pellet suspended in 2× protein sample buffer normalized based on the OD_600_ readings: that is, the 1-ml supernatant fraction derived from a culture with an OD_600_ of 2 was suspended in 100 μl sample buffer. The supernatant and cell-associated fractions were boiled for 20 min and centrifuged for 5 min at 17,000 × *g*, and 10 μl of each sample was separated on a 10% polyacrylamide (PAA) gel. Proteins were transferred to polyvinylidene difluoride (PVDF) membranes, and Western blots were performed either using an HRP-conjugated anti-His antibody (Sigma) at a 1:5,000 dilution, an anti-L6 ribosomal protein primary antibody at a 1:20,000 dilution, or the Ami-specific polyclonal antibody R4 (11) at a 1:5,000 dilution and an HRP-conjugated anti-rabbit secondary antibody (Cell Signaling Technology) at a 1:10,000 dilution. For the detection of the native InlB protein, strains 10403S and EGD were grown overnight in either BHI or BHI–0.2% activated charcoal at 37°C and the cell and supernatant fractions were prepared as described above. The InlB protein was detected using the mouse monoclonal InlB antibody R4.6 (11) at a 1:1,000 dilution and an HRP-conjugated anti-mouse secondary antibody (Cell Signaling Technology) at a 1:10,000 dilution. Blots were developed by the enhanced chemiluminescence (ECL) method, the signal was captured using an ECL Hyperfilm (GE Healthcare), and the film was developed with an automated developer (AGFA-Healthcare N.V.), or the signal was directly detected using a ChemiDoc Tough imager (Bio-Rad).

### WTA isolation and detection by alcian blue-silver staining.

The cells from 45-ml overnight cultures of the indicated L. monocytogenes strains were harvested by centrifugation for 10 min at 6,000 × *g*. The pellet was washed with 20 ml 20 mM sodium acetate (pH 4.6) buffer and suspended in 800 μl of the same buffer. The cell suspension was transferred to a tube containing an equal volume of 0.1-mm glass beads. The bacteria were lysed in a FastPrep machine (MP Bioscience) using three 1-min runs with 2-min incubations on ice between runs. The samples were centrifuged at 200 × *g* for 1 min to sediment the glass beads, and the supernatant was transferred to a fresh tube. The glass beads were suspended in 750 μl 20 mM sodium acetate (pH 4.6) buffer centrifuged as before, and the supernatants were combined. The cell walls in the supernatant fractions were harvested by centrifugation at 17,000 × *g* for 5 min and suspended in a final volume of 1 ml 20 mM sodium acetate buffer (pH 4.6) containing 4% SDS. The cell wall suspensions were boiled for 30 min and transferred to a fresh tube, and the volume was made up to 10 ml with 20 mM sodium acetate (pH 4.6) buffer. The samples were centrifuged at 6,000 × *g* for 10 min and washed twice more with the same buffer. After the final centrifugation step, the pellet was then suspended in 800 μl of the same buffer and centrifuged at 17,000 × *g* for 5 min. The pellet was suspended in 20 mM sodium acetate (pH 4.6) normalized based on the OD_600_ reading of the overnight culture: that is, 1 ml of a culture with an original OD_600_ of 1 was suspended in 10 μl buffer. To liberate the WTA, NaOH was added to a final concentration of 0.1 M and the samples were incubated at 65°C for 2 h. The sample was centrifuged for 10 min at 17,000 × *g*, and the supernatant was transferred to a fresh tube and stored at −20°C. To analyze the WTA, 10 μl of NaOH-extracted WTA samples was mixed with 10 μl of 2 M sucrose prepared in Tris-borate-EDTA (TBE) buffer, and then 5-μl aliquots were loaded onto a 15% TBE–PAA gel and run at 100 V for 1 h in 0.5× TBE buffer. The WTA was visualized using an alcian blue-silver staining method, which was performed with minor modifications as described previously (42). Briefly, the PAA gels were immersed in 0.5% alcian blue solution made up in 1% acetic acid and incubated with moderate shaking for 1 h at room temperature. Excess alcian blue was removed by rinsing the gel several times with ddH_2_O and then incubation with ddH_2_O for 1 h. Following this, the gel was fixed with 40% methanol and 10% acetic acid. Destained and fixed TBE-PAA gels were washed again in ddH_2_O, and the gel was subsequently overlaid with oxidation solution (3.4 mM potassium dichromate, 3.2 mM nitric acid) and incubated for 10 min with moderate shaking. The gels were washed twice with ddH_2_O and following this immersed in 12 mM silver nitrate solution for 20 min and then washed once briefly in ddH_2_O. The gel was overlaid with developing solution (0.28 M sodium carbonate and 6 mM formaldehyde) for 10 s, and then the solution was discarded and fresh developing solution added until a brown precipitate was observed. To stop further coloration, the developing solution was removed and a 5% acetic acid stop solution added.

### Protein expression and purification.

Strains BL21(DE3)/pVL847 (ANG2890) and BL21(DE3)/pVL847-GW_Ami_ (ANG3181) were used for the expression and purification of the His-MBP and His-MBP-GW_Ami_ proteins. Protein expression was induced with 0.5 mM IPTG (isopropyl-β-d-thiogalactopyranoside) at 16°C overnight, and protein purification by Ni-NTA and size exclusion chromatograph was essentially performed as previously described (43), with the exception that EDTA-free protease inhibitors (Roche) and 10 mM imidazole were added to the lysis buffer and 20 mM Tris (pH 7.5)–200 mM NaCl–5% glycerol buffer was used for the size exclusion chromatography step. Following size exclusion chromatography, the protein-containing fractions were pooled and concentrated using a 10-kDa-cutoff Amicon Centricon concentrator (Millipore) and the final protein concentration was determined using the bicinchoninic acid (BCA) assay kit (Pierce). Protein samples were snap-frozen in N_2_ and then stored at −80°C.

### Peptidoglycan purification.

Peptidoglycan was purified from the strains 10403S and 10403S Δ*tagO1-2*_sup_. For the WT strain, an overnight culture was used to inoculate 1 liter of BHI medium to an OD_600_ of 0.06, and the culture was grown at 37°C until it reached an OD_600_ of 1. For the 10403S Δ*tagO1-2*_sup_ strain, two 1-liter cultures were grown in the same way to an OD_600_ of 0.5 to 0.6. From this point on, all cultures were treated the same. Peptidoglycan was purified as previously described (44). Purified peptidoglycan sacculi were lyophilized prior to the hydrofluoric acid (HF) treatment, their dry weight was determined, and suspensions at the indicated concentration in milligrams per milliliter were prepared and used in binding assays. To remove wall teichoic acid, the lyophilized peptidoglycan material from strain 10403S was suspended in 48% HF at a concentration of 2.5 mg/ml. The suspension was incubated at 4°C with moderate agitation for 48 h. Following this, the peptidoglycans were harvested by centrifugation, washed twice with 25 ml ddH_2_O, twice with 25 ml 100 mM Tris (pH 7.5), and twice more with 25 ml ddH_2_O. The peptidoglycan was suspended in a final volume of 5 ml 100 mM (NH_4_)_2_CO_3_. To this suspension, 200 U of alkaline phosphatase (Sigma) was added, and the suspension was incubated at 37°C for 16 h on a rotator wheel. The samples were subsequently boiled for 5 min and then washed twice with 25 ml ddH_2_O. The peptidoglycan was again lyophilized, the dry weight was determined, and suspensions at the indicated concentration in milligrams per milliliter were prepared and used in binding assays.

### Binding assays with whole cells or purified peptidoglycan and purified MBP fusion proteins.

Bacteria from a 50-ml overnight culture of L. monocytogenes strain 10403S or 10403S Δ*tagO1-2*_sup_ were harvested by centrifugation, washed once with 20 ml 50 mM Tris (pH 8), and subsequently suspended in the same buffer to an OD_600_ of 10, 1, or 0.5. Protein binding assays were set up in 250-μl reaction and consisted of 200 μl of this cell suspension and 50 μl of purified His-MBP or His-MBP-GW_Ami_ protein at a concentration of 40 μM, giving a final protein concentration of 8 μM in the reaction mixture. Control reaction mixtures that lacked cells (no-cell reactions) consisted of 200 μl 50 mM Tris (pH 8) buffer plus 50 μl of protein solution. The reactions were vortexed then incubated at room temperature for 15 min. Following this, the samples were centrifuged for 10 min at 17,000 × *g* to pellet the bacteria and any bound protein. The supernatant fraction containing the unbound protein was moved to a fresh tube, the same volume of 2× protein sample buffer was added, 10 μl of these samples was analyzed on a 10% PAA gel, and proteins were visualized by Coomassie staining. The binding assays with purified peptidoglycan were set up in a similar manner. Each 250-μl reaction mixture consisted of 200 μl purified peptidoglycan suspensions (either before or after HF treatment) at the indicated concentration in 50 mM Tris (pH 8) buffer and 50 μl of a 40 μM His-MBP or His-MBP-GW_Ami_ protein solution. Control reaction mixtures that lacked peptidoglycan (no-PG reactions) contained only 200 μl of 50 mM Tris (pH 8) buffer and 50 μl of the protein solutions. The reaction mixtures were vortexed and incubated at room temperature on a rotator wheel for 15 min and subsequently prepared and analyzed as described above for whole-cell binding assays.

### Whole-genome sequencing and bioinformatics analysis.

Genome sequencing of L. monocytogenes strains 10403S, 10403S Δ*ltaS*_sup_ (ANG2337), and 10403S Δ*tagO1-2*_sup_ (ANG2350) was performed by microbesNG (http://microbesng.uk) using an Illumina MiSeq platform and 2× 250-bp paired-end reads. The CLC genomics workbench software package (Qiagen) was used to map the reads to the 10403S reference genome sequence (RefSeq accession no. NC_017544.1), and high-confidence and high-frequency (>85%) changes are listed in Table S3 for the different strains. To determine the conservation of the *gtlA* gene among other Listeria species, a synteny analysis was performed using the SyntTax web server (http://archaea.u-psud.fr/synttax/) and all available sequenced Listeria genomes (45)

### Nucleotide sequence accession number.

The genome sequence data have been deposited in the European Nucleotide Archive under accession no. PRJEB11525 (http://www.ebi.ac.uk/ena/data/view/PRJEB11525).

## RESULTS

### Identification of a type I LTA glycosyltransferase in L. monocytogenes.

**FIG 2:**
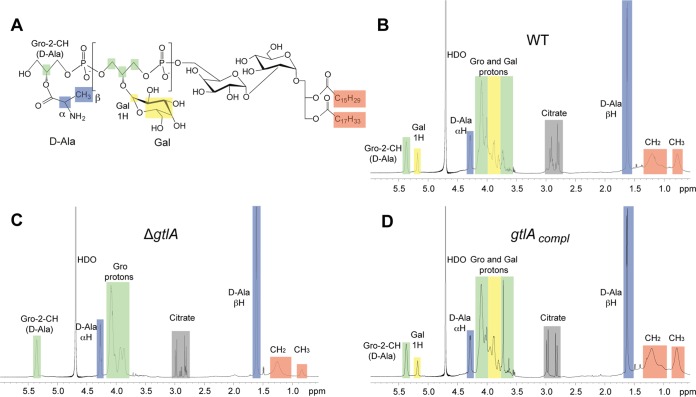
Chemical structure and NMR analysis of LTA isolated from WT L. monocytogenes strain 10403S, the *gtlA* (*lmo0933*) mutant, and the complementation strain. (A) Chemical structure of type I LTA synthesized by L. monocytogenes. Colored boxes indicate nonexchangeable protons in the LTA, which are referred to in the text and shaded in the same colors in the NMR spectra. Peaks highlighted in gray are derived from residual citrate, a buffer component used during the LTA purification process. (B to D) NMR spectra of LTA samples derived from L. monocytogenes strains (B) 10403S (WT), (C) 10403S Δ*gtlA* (Δ*gtlA*), and (D) 10403S Δ*gtlA*/pPL3e-*gtlA* (*gtlA_compl_*). The signals derived from the nonexchangeable protons were assigned to the different LTA components based on previously published spectra (39–41). The spectra are representative of at least three independent experiments.

Glycosyltransferases (GTs) belonging to the GT2 family of glycosyltransferase enzymes have been shown to catalyze, in the presence of divalent cations, the transfer of sugar moieties from a nucleotide-activated precursor onto the lipid carrier molecule C_55_-P. Therefore, we hypothesized that the cytoplasmic GT involved in the glycosylation process of type I LTA is a GT2 family member (Fig. 1A). To identify such enzymes in L. monocytogenes, a BLAST search was performed using the E. coli ArnC protein, which transfers 4-amino-4-deoxyl-4-formamido-l-arabinose (l-Ara4FN) from UDP-β-l-Ara4FN onto C_55_-P (34), as the query sequence. Two proteins, Lmo0933 and Lmo2550, with E values of 1e−35 and 1e−42, respectively, were identified as the closest ArnC homologues in the L. monocytogenes EGD-e genome, corresponding to proteins LMRG_02032 and LMRG_01697 in L. monocytogenes strain 10403S, which was used in this study. Recently, it has been shown that Lmo2550 is required for the modification of WTA with GlcNAc residues (46), and therefore our further analysis focused on Lmo0933. An in-frame deletion was generated in *lmo0933*, which as discussed below was renamed *gtlA*, yielding strain 10403S Δ*gtlA*. In addition a complementation strain, 10403S Δ*gtlA*/pPL3e-*gtlA*, was constructed, in which *gtlA* was expressed from its native promoter in the chromosomal integration vector pPL3e. As additional controls, the empty pPL3e vector was also introduced into the WT and the Δ*gtlA* mutant strains. To assess if deletion of *gtlA* affects LTA production, we initially performed a Western blot analysis on cell extracts prepared from the different strains using a polyglycerol phosphate-specific monoclonal LTA antibody. As shown in Fig. 1B, a polyglycerol phosphate polymer was produced by all strains. However, the signal obtained from the extract prepared from the *gtlA* mutant strain was noticeably stronger than that obtained from extracts prepared from the WT strain, indicating structural changes in the LTA polymer. This phenotype could be complemented by the introduction of the vector pPL3e-*gtlA* but not the empty vector (Fig. 1B). To determine the exact nature of these structural changes, LTA was purified from the wild-type (WT) L. monocytogenes strain 10403S, as well as the *gtlA* (Δ*gtlA*) mutant and the complementation strain 10403S Δ*gtlA*/pPL3e-*gtlA* (*gtlA_compl_*). Labile protons were exchanged with deuterons by repeated suspension and freeze-drying of the LTA samples in D_2_O, and the samples were subsequently analyzed by 1D ^1^H NMR (Fig. 2). The LTA isolated from the WT strain yielded the expected NMR spectrum, and peaks obtained from the nonexchangeable protons could be assigned to the different LTA components using previously published spectra as references (39–41). Specifically, peaks derived from the CH_2_ and CH_3_ groups within the fatty acids (highlighted in orange), the CH_2_ groups from the glycerol backbone (highlighted in green), the CH_3_ and CH groups from the d-Ala modification (highlighted in blue), and CH and CH_2_ groups from the galactose substitutions (highlighted in yellow) could be assigned as indicated in Fig. 2. The galactose-specific peaks were absent in the NMR spectrum from the LTA sample obtained from strain 10403S Δ*gtlA*. The NMR spectrum for the LTA derived from strain 10403S Δ*gtlA*/pPL3e-*gtlA* (*gtlA_compl_*) was nearly identical to that obtained from the WT strain, showing that this defect was complemented by the reintroduction of *gtlA* (Fig. 2). These data suggest that Lmo0933 is likely the cytoplasmic glycosyltransferase required for the glycosylation of type I LTA, and hence Lmo0933 was renamed GtlA, for glycosyltransferase LTA A.

### LTA glycosylation does not affect LTA chain length or d-alanylation.

**FIG 3:**
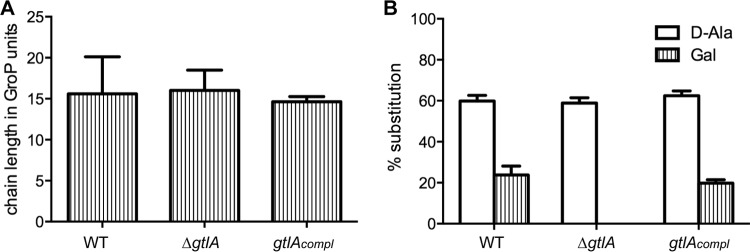
Determination of the LTA chain length and percentages of d-Ala and Gal substitutions. LTA was extracted from strains 10403S (WT) and 10403S Δ*gtlA* (Δ*gtlA*) and the complementation strain 10403S Δ*gtlA*/pPL3e-*gtlA* (*gtlA_compl_*) and analyzed by NMR as shown in Fig. 2. The peaks were integrated and (A) the chain length and (B) the percentages of d-Ala and Gal substitutions were determined as described in Materials and Methods. The average values and standard deviations from three independent experiments are plotted. Statistical significance was determined using a two-tailed unpaired Student *t* test, and only a statistical difference with a *P* value of <0.05 was observed for the percentage of Gal substitution between the *gtlA* mutant and the WT or complementation strains. Otherwise the lowest *P* value was *P* = 0.2, indicating that there is no statistical significance difference in LTA chain length and percentage of d-Ala modifications between all three strains or the percentage of Gal substitution between the WT and the complementation strain.

To assess if the absence of the galactosyl modifications affects the chain length of LTA and/or the amount of d-alanine modification, the peaks in the NMR spectra were integrated and the chain length and percentage of alanine and galactose substitutions were calculated as described in Materials and Methods. The NMR spectra from three independently isolated LTA samples were analyzed for each strain and the average values and standard deviations plotted (Fig. 3). The LTA isolated from the WT 10403S strain had an average chain length of 15.6 ± 4.5 GroP repeating units modified with 59.9% ± 2.7% d-Ala and 23.8% ± 4.3% Gal substitutions, respectively. No statistically significant difference in the LTA chain lengths and percentages of d-alanine substitution was detected for the LTA isolated from strain 10403S Δ*gtlA* and the complementation strain compared to the sample derived from the WT strain (Fig. 3). Furthermore, the amount of galactosyl substitutions could be restored to wild-type levels in the complementation strain (Fig. 3). These data indicate that the absence of galactosyl residues affects neither the LTA chain length nor the amount of d-alanylation.

### Phenotypic characterization of a *gtlA* mutant strain.

**FIG 4:**
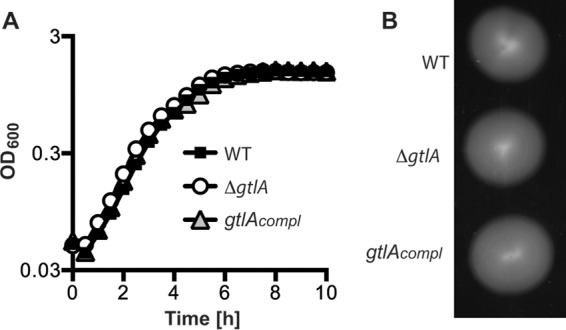
Growth and motility of the L. monocytogenes strain WT, *gtlA* mutant, and complementation strain. (A) Bacterial growth curve. L. monocytogenes strains 10403S (WT) and 10403S Δ*gtlA* (Δ*gtlA*) and the complementation strain 10403S Δ*gtlA*/pPL3e-*gtlA* (*gtlA_compl_*) were grown in BHI medium in 96-well plates at 37°C, and OD_600_ readings were determined at the indicated time points and plotted. (B) Plate motility assay. The same L. monocytogenes strains as in panel A were stab inoculated on low-agar (0.3%) BHI plates and incubated at 30°C for 18 h. The experiment was performed in triplicate, and a representative result is shown.

A phenotypic analysis of the 10403S Δ*gtlA* strain revealed that the galactosyl modification on LTA is not required for growth in broth culture or flagellum-based motility (Fig. 4). In previous work, it has been shown that an L. monocytogenes strain lacking d-Ala modifications on LTA is more sensitive toward cationic antimicrobial peptides and the antibiotic nisin (29). To test if the absence of galactose modifications on LTA similarly affects the antibiotic susceptibility of strain 10403S Δ*gtlA*, the MIC toward nicin was tested. No difference was observed, and the WT and mutant strains had identical MICs of 12.5 μg/ml. These data indicate that in contrast to the d-alanine modifications on LTA, galactose modifications do not play a role in cationic antimicrobial resistance. Identical MIC values for the cell wall-targeting antibiotics ampicillin and penicillin G of 0.125 μg/ml and 3.125 μg/ml, respectively, were also observed for the WT and *gtlA* mutant strains, suggesting that the mutant strain does not have any major defects in its peptidoglycan structure.

### GW-domain-containing proteins are retained in the cell wall of L. monocytogenes mutants lacking LTA modifications.

**FIG 5:**
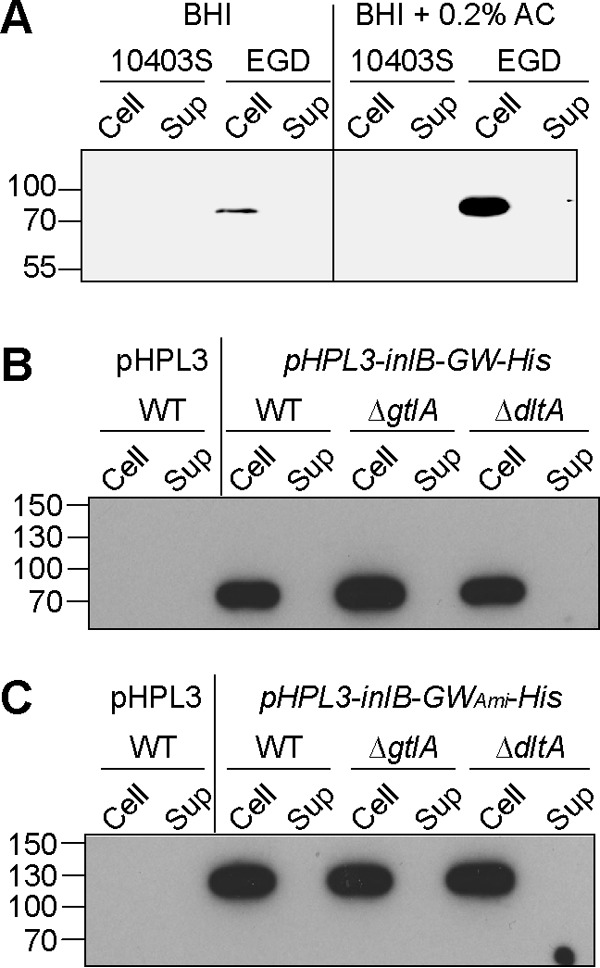
Western blot analysis for the detection of InlB or His-tagged GW domain reporter proteins in the cell or supernatant fraction of L. monocytogenes strains lacking LTA modifications. (A) Cell and supernatant samples were prepared from overnight cultures of L. monocytogenes strains 10403S and EGD grown in BHI or BHI–0.2% activated charcoal (BHI + 0.2% AC) medium, and the InlB protein was detected by Western blotting. Ten microliters of the cell-associated protein samples (Cell) or supernatant samples (Sup) was separated on 10% PAA gels, and the InlB protein was detected using an InlB-specific antibody at a 1:1,000 dilution and an HRP-conjugated anti-mouse antibody at a 1:10,000 dilution, respectively. (B and C) Cell and supernatant samples were prepared from overnight cultures of L. monocytogenes strains 10403S (WT), 10403S Δ*gtlA* (Δ*gtlA*), and 10403S Δ*dltA* (Δ*dltA*) containing (B) plasmid pHPL3-*inlB*-GW-His (InlB-GW-His) or (C) plasmid pHPL3-*inlB-amiGW*-His (InlB-GW_Ami_-His) and subsequently analyzed by Western blotting as described in panel A using an HRP-conjugated anti-His antibody at a 1:1,000. The positions of the protein molecular mass markers are indicated on the left in kilodaltons. Representative blots from three independent experiments are shown.

While an L. monocytogenes strain lacking glycosyl modification on LTA did not show any obvious growth phenotypes, previous work has indicated that LTA serves as binding receptor for the GW-domain-containing cell wall proteins InlB and Ami (12). Therefore, we next wanted to test if this modification is required for the retention of noncovalently attached cell wall proteins. The enzymes required for the synthesis of the LTA backbone (25), d-Ala substitutions (29), and Gal substitutions have now been identified, and this allowed us to address this question using the different mutant strains. In previous work, it has been shown that during exponential growth, a large amount of the InlB protein is retained in strain EGD in a GW-domain-dependent manner within the cell wall fraction, with some of the protein released into the supernatant fraction (11). Using a similar fractionation experiment and an InlB-specific antibody, we found that after overnight growth, the InlB protein is exclusively found in the cell wall fraction of strain EGD (Fig. 5A). Here it is of note that it was recently found that strain EGD contains a pfrA* allele leading to increased production of many virulence factors (47). As the different LTA mutant strains have been constructed in the 10403S strain background, we next attempted to assess the InlB localization in strain 10403S. However, even following growth in BHI–0.2% activated charcoal medium, which is known to induce the expression of PrfA-dependent virulence proteins such as InlB, we were unable to detect significant amounts of the native InlB protein in strain 10403 (Fig. 5A). Hence we could not assess the localization of the InlB protein when expressed from the native promoter in strain 10403S. Therefore, we next constructed plasmids pHPL3-*inlB*-GW-His and pHPL3-inlB-GW_Ami_-His for the expression of C-terminally His-tagged InlB (referred to as InlB-GW-His) as well as a variant of InlB in which its three native GW domains were replaced with the eight GW domains of the Ami protein (referred to as InlB-GW_Ami_-His) from the strong and constitutive hyper spac promoter. These single-site integration vectors were initially introduced into the WT L. monocytogenes strain 10403S and strains 10403S Δ*gtlA* and 10403S Δ*dltA*, lacking Gal and d-Ala modifications on their LTA, respectively. The empty vector pHPL3 was also integrated into the chromosome of strain 10403S, to yield a negative-control strain. All strains were grown overnight in liquid culture, a cell fractionation experiment was performed, and the His-tagged proteins were subsequently detected by Western blotting in the pellet (cell wall-associated proteins) and supernatant fraction. As expected, no band was detected in samples prepared from the 10403S control strain containing the empty vector (Fig. 5B and C). Clear bands for the InlB-GW-His and InlB-GW_Ami_-His reporter proteins could be detected in all other samples. More importantly, the reporter proteins were detected in the cell pellet fraction for all strains, and no signal was detected in the supernatant fractions, indicating that neither the d-alanine nor the Gal modification on LTA is required for the retention of GW-domain-containing proteins within the cell wall of L. monocytogenes (Fig. 5B and C).

### GW domain proteins are retained in the cell wall of L. monocytogenes mutants lacking the LTA polymer.

**FIG 6:**
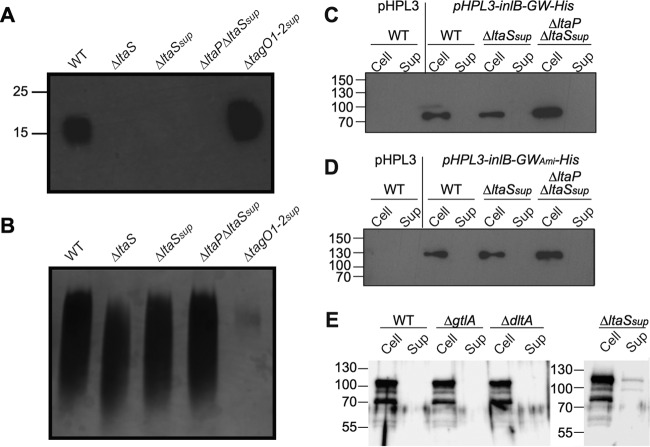
LTA, WTA, and Western blot analysis for the detection of His-tagged GW domain reporter proteins and Ami protein in the cell or supernatant fraction of L. monocytogenes strains lacking the LTA polymer. (A) Detection of LTA by Western blotting. Extracts from strains 10403S (WT), 10403S Δ*ltaS* (Δ*ltaS*), 10403S Δ*ltaA*_sup_ (Δ*ltaS_sup_*), 10403S Δ*ltaP* Δ*ltaS*_sup_ (Δ*ltaP*Δ*ltaS_sup_*), and 10403S Δ*tagO1-2*_sup_ (Δ*tagO1-2_sup_*) were prepared and separated on 15% polyacrylamide gels as described in Materials and Methods. The LTA polymer was detected by Western blotting using a polyglycerol phosphate-specific monoclonal antibody; positions of protein standards (in kilodaltons) are indicated on the left-hand side. (B) Detection of WTA by alcian blue and silver staining. WTA was isolated from the same L. monocytogenes strains as in panel A, separated on a native polyacrylamide gel, and WTA visualized as described in Materials and Methods by an alcian blue-silver staining procedure. (C and D) Cell and supernatant samples were prepared from overnight cultures of L. monocytogenes strains 10403S (WT), 10403S Δ*ltaS*_sup_, and 10403S Δ*ltaP* Δ*ltaS*_sup_ containing (C) plasmid pHPL3-*inlB*-His (InlB-GW-His) or (D) plasmid pHPL3-*inlB-amiGW*-His (InlB-GW_Ami_-His), and His-tagged proteins were detected by Western blot analysis as in Fig. 4. (E) Western blot analysis for the detection of Ami in the cell or supernatant fraction of L. monocytogenes strains lacking LTA or its modifications. Cell and supernatant samples were prepared from overnight cultures of L. monocytogenes strains 10403S (WT), 10403S Δ*gtlA* (Δ*gtlA*), 10403S Δ*dltA* (Δ*dltA*) and 10403S Δ*ltaS*_sup_ (Δ*ltaS_sup_*.). Ten microliters of the cell-associated protein samples (Cell) or supernatant samples (Sup) was separated on 10% PAA gels, and the Ami protein was detected using a polyclonal rabbit antibody at 1:5,000. The positions of the protein molecular mass markers are indicated on the left in kilodaltons. Representative results from three independent experiments are shown.

Next, we investigated whether or not the GW domain proteins would bind directly to the polyglycerol phosphate backbone of LTA. Strain 10403S Δ*ltaS*, which produces the GroP-glycolipid intermediate but is unable to polymerize the LTA backbone chain, was constructed in a previous study (25). This strain has a severe growth defect at 37°C, and bacteria tend to lyse (25). This makes this strain difficult to handle and transform with plasmids, so a suppressor strain, 10403S Δ*ltaS*_sup_, with improved growth was generated by repeated passage at 37°C. The 10403S strain used in this study has four high-frequency and high-quality single nucleotide polymorphisms (SNPs), compared to the genome sequence of strain 10403S deposited under RefSeq accession no. NC_017544.1 (see Table S3 in the supplemental material). Whole-genome sequencing revealed that strain 10403S Δ*ltaS*_sup_ has eight additional SNPs compared to our WT strain. Two of these SNPs are in intergenic regions, and six are within coding regions, and of these two are within genes coding for enzymes that are directly related to cell wall synthesis, namely the peptidoglycan hydrolase Iap (or p60) and the d-glutamyl-l-m-Dpm peptidase P45. The nucleotide substitution in *iap* does not lead to an actual amino acid substitution, and it is therefore unclear if this SNP affects the protein function (see Table S3 in the supplemental material). On the other hand, the single nucleotide insertion in the P45 gene leads to a frameshift mutation and likely inactivation of the enzyme, potentially causing changes in the peptidoglycan structure. In addition to these SNPs, strain 10403S Δ*ltaS*_sup_ has the engineered large deletion in the *ltaS* gene and was therefore still unable to produce the LTA polymer, as assessed by Western blot analysis (Fig. 6A). Of note, strain 10403S Δ*ltaS*_sup_ was still able to produce the other cell wall polymer, WTA (Fig. 6B). In order to also prevent the synthesis of the GroP-glycolipid intermediate, the *ltaP* gene was then deleted, yielding the double mutant 10403S Δ*ltaP* Δ*ltaS*_sup_ strain, which was, as expected, LTA negative and WTA positive (Fig. 6A and B). Next, plasmids pHPL3-*inlB-GW*-His and pHPL3-*inlB*-GW_Ami_-His were introduced into strains 10403S Δ*ltaS*_sup_ and 10403S Δ*ltaP* Δ*ltaS*_sup_, and the cellular location of the reporter proteins was assessed by a cell fractionation and Western blot experiment. Surprisingly, the signal for the reporter protein was again detected in the pellet fraction in both strains, revealing that GW-domain-containing proteins can be retained within the cell wall, even in a strain completely lacking the LTA polymer (Fig. 6C and D). To investigate this further, we also assessed the localization of the Ami protein in wild-type as well as mutant L. monocytogenes strains lacking LTA or its modification. As observed for the InlB constructs, the native Ami protein was primarily found within the cell wall fraction but was absent from the supernatant fraction (Fig. 6E). Taken together, these data suggest that the LTA polymer is not the only cell wall receptor for GW-domain-containing proteins in L. monocytogenes.

### Assessing the contribution of WTA in the retention of GW-domain-containing cell wall proteins.

**FIG 7:**
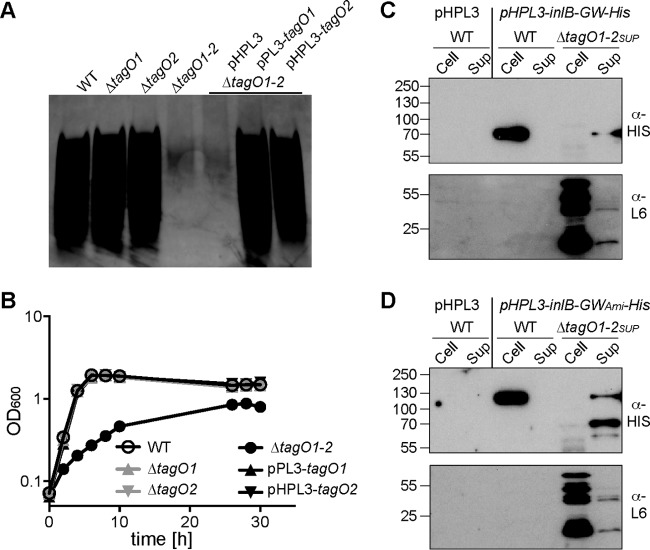
Growth, WTA detection, and Western blot analysis for the detection of His-tagged GW domain reporter proteins in the cell or supernatant fraction of L. monocytogenes strains lacking the WTA polymer. (A) Detection of WTA by alcian blue and silver staining. WTA was isolated from the indicated L. monocytogenes strains, separated on a native polyacrylamide gel, and WTA visualized as described in Materials and Methods by an alcian blue-silver staining procedure. (B) Bacterial growth curve. Overnight cultures of the WT L. monocytogenes strain 10403S, the single *tagO1* and *tagO2* mutants, and the double *tagO1 tagO2* mutant as well as the *tagO1-2* double mutant strain containing the complementation vector pPL3-*tagO1* or pHPL3-*tagO2* were diluted to a starting OD_600_ of 0.07, and cultures were incubated at 37°C. Bacterial growth was monitored by determining OD_600_ readings at the indicated time points. Representative graphs of two independent experiments are shown. (C and D) Cell and supernatant (Sup) samples were prepared from overnight cultures of L. monocytogenes strains 10403S (WT) and 10403S Δ*tagO1-2*_sup_ containing (C) plasmid pHPL3-*inlB*-His (*pHPL3-InlB-His*) or (D) plasmid pHPL3-*inlB*-GW_Ami_-His. Strain 10403S (WT) containing the empty vector pHPL3 was used as the negative control. For the top panel, His-tagged proteins were detected as described in Fig. 5 using an HRP-conjugated anti-His antibody, and in the bottom panel, membranes were incubated with an anti-L6 ribosomal protein primary antibody and an HRP-conjugated antirabbit secondary antibody. The positions of protein molecular mass markers are indicated on the left of each panel in kilodaltons. Representative blots from three independent experiments are shown.

The two other major cell wall polymers in L. monocytogenes are WTA and peptidoglycan. WTA synthesis is initiated in the cytoplasm of the cell by a reaction catalyzed by the TagO enzyme. L. monocytogenes contains two *tagO* genes, *lmo0959* (or LMRG_02058 in strain 10403S and referred to as *tagO1*) and *lmo2519* (or LMRG_01729 in strain 10403S and referred to as *tagO2*). A WTA depletion strain has been described recently in the EGD-e strain background, in which both *tagO* genes were deleted and the *tagO1* gene was expressed in *trans* from an IPTG-inducible promoter (21). The strain showed a severe growth defect. In order to assess the contribution of WTA in the retention of GW-domain-containing proteins, we consecutively deleted both *tagO* genes in the 10403S strain background, yielding strain 10403S Δ*tagO1-2*. As expected, the double *tagO* mutant strain 10403S Δ*tagO1-2* lacked WTA (Fig. 7A) but was still able to produce the LTA polymer (Fig. 6A). This strain exhibited a severe growth defect, and both WTA production and the growth defect could be complemented by expression of either of the *tagO* genes (Fig. 7B). Next, we set out to assess the contribution of WTA in the cell wall retention of GW-domain-containing proteins. However, strain 10403S Δ*tagO1-2* was very difficult to grow and the bacteria were prone to lyse, so we were not able to introduce plasmids pHPL3-*inlB*-GW-His and pHPL3-*inlB*-GW_Ami_-His for expression of the reporter proteins into this strain. Only after strain 10403S Δ*tagO1-2* was passaged several times in broth culture and the suppressor strain 10403S Δ*tagO1-2*_sup_ with improved growth was obtained was it possible to introduce plasmids pHPL3-*inlB*-GW-His and pHPL3-*inlB*-GW_Ami_-His. As determined by whole-genome sequencing, strain 10403S Δ*tagO1-2*_sup_ contained, besides the engineered deletions in the *tagO* genes, a single SNP. This SNP is in *plsX* coding for an acyl-acyl carrier protein (ACP) phosphate acyltransferase, which is involved in membrane lipid biosynthesis (48). Strain 10403S Δ*tagO1-2*_sup_ was, as expected, unable to produce WTA but was still able to produce LTA (Fig. 6A and B). 10403S Δ*tagO1-2*_sup_-derived strains were, however, still very fragile, as exemplified by the following observation: boiling wild-type 10403S bacteria in protein sample buffer does not lead to cell lysis and release of cytoplasmic proteins, such as the ribosomal protein L6 (Fig. 7C and D), yet the same treatment leads to cell lysis and release of cytoplasmic proteins in strain 10403S Δ*tagO1*-2_sup_ (Fig. 7C and D). With this caveat in mind, we still went ahead and performed a cell fractionation experiment aimed to detect the InlB-GW-His and InlB-GW_Ami_-His proteins in the cell pellet or supernatant fraction. Only very small amounts of full-length proteins could be detected, and these were detected predominately in the supernatant fraction (Fig. 7C and D). These results indicate that GW domain proteins might be retained in the cell wall by binding to the WTA structure. However, given the severe growth defect and fragile nature of the WTA-negative strain, these data need to be interpreted with caution. As described below, we therefore set up an alternative method to assess the contribution of WTA as the binding receptor for GW-domain-containing proteins.

### The purified MBP-GW_Ami_ fusion protein can bind to L. monocytogenes cells lacking WTA.

**FIG 8:**
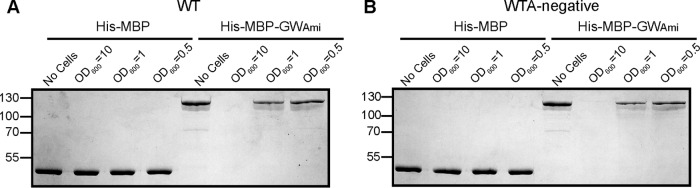
Binding of purified His-MBP-GW_Ami_ to whole cells of wild-type and WTA-deficient L. monocytogenes cells. Purified His-MBP or His-MBP-GW_Ami_ protein was incubated in the absence of cells or in the presence of (A) 10403S (WT) cell suspensions or (B) 10403S Δ*tagO1-2*_sup_ (WTA-negative) cell suspensions. “No cells” indicates control samples where the proteins were incubated without cells, and samples where the protein was incubated with bacterial suspensions are labeled “OD_600_ = 10,” “OD_600_ = 1,” or “OD_600_ = 0.5,” depending on cell densities. Cells and bound protein were removed by centrifugation, and then an aliquot of the supernatant fraction was analyzed by SDS-PAGE and proteins visualized by Coomassie staining. The His-MBP and His-MBP-GW_Ami_ proteins have predicted sizes of 45 kDa and 118 kDa, respectively. The positions of protein molecular mass markers are indicated on the left in kilodaltons. Representative gels from three independent experiments are shown.

Purified proteins containing GW domains can bind to WT L. monocytogenes cells when added externally (11). We now wanted to test if such reporter proteins could also bind to L. monocytogenes cells lacking WTA. To this end, an E. coli plasmid for the expression of an N-terminally His-tagged maltose binding protein (His-MBP) fused to the Ami GW domains was constructed. The His-MBP-GW_Ami_ fusion protein as well as the His-MBP control protein were expressed in E. coli and purified by Ni-affinity and size exclusion chromatography. Of note, a similar construct for expression of the His-MBP-GW_InlB_ fusion protein was made; however, the protein aggregated during the purification process and could not be used for further analysis. Next, the purified His-MBP and His-MBP-GW_Ami_ fusion proteins were incubated with cell suspensions derived from strain 10403S (WT) or the WTA-deficient strain 10403S Δ*tagO1-2*_sup_ (WTA negative). Suspensions of different densities corresponding to OD_600_ readings of 10, 1, or 0.5 were used, and as a control, the purified proteins were also incubated in the absence of cells (“no-cell” samples). The samples were then centrifuged to pellet the bacteria and any bound protein, and then the supernatant fraction was removed and analyzed by SDS-PAGE, and any unbound protein was visualized by Coomassie staining. As expected, the His-MBP control protein did not bind to the bacterial cells and the protein was recovered in the supernatant fraction in all samples, regardless of whether the protein was incubated in the absence of cells or in the presence of WT or 10403S Δ*tagO1-2*_sup_ cells (Fig. 8). Also as expected, the His-MBP-GW_Ami_ bound and pelleted with WT bacteria and was completely removed from the supernatant fraction when using high-density cell suspension and partially removed when using lower cell density suspensions (Fig. 8A). A similar result was obtained when 10403S Δ*tagO1-2*_sup_ cell suspensions were used, as the reporter protein pelleted with the bacteria and was absent from the supernatant fraction when high-density cell suspensions were used or was only partially recovered in the supernatant fraction when lower-density cell suspensions were used (Fig. 8B). These results suggest that WTA is not required for the cell wall binding of GW domain proteins.

### The purified MBP-GW_Ami_ fusion protein binds to purified peptidoglycan.

**FIG 9:**
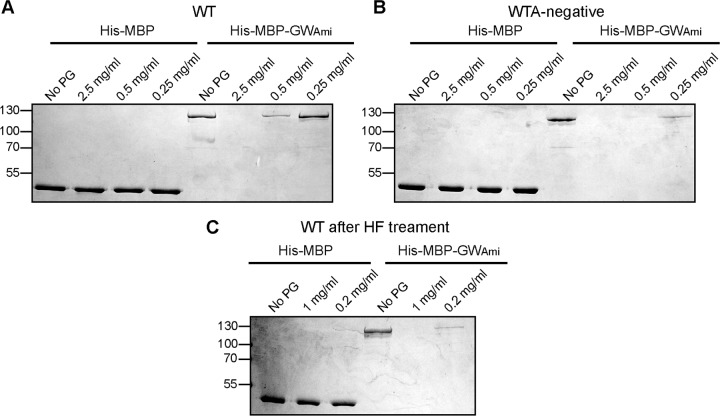
Binding of His-MBP-GW_Ami_ to purified peptidoglycan isolated from wild-type or WTA-deficient L. monocytogenes cells. (A and B) Peptidoglycan isolated from strain 10403S (WT) or the WTA-deficient strain 10403S Δ*tagO1-2*_sup_ (WTA negative) was suspended at the indicated concentration prior to HF treatment and incubated with purified His-MBP or His-MBP-GW_Ami_ protein. “No PG” indicates control samples where the proteins were incubated without peptidoglycan. Peptidoglycan and bound protein were removed by centrifugation, an aliquot of the supernatant fraction was analyzed by SDS-PAGE, and the proteins were visualized by Coomassie staining. (C) Peptidoglycan isolated from strain 10403S (WT) was suspended at the indicated concentration after HF treatment to chemically remove WTA and incubated with purified His-MBP or His-MBP-GW_Ami_ protein and processed as described above. The His-MBP or His-MBP-GW_Ami_ proteins have predicted sizes of 45 kDa and 118 kDa, respectively. The positions of protein molecular mass markers are indicated on the left in kilodaltons. Representative gels from three independent experiments are shown.

To investigate whether GW-domain-containing proteins might directly bind to peptidoglycan, this cell wall polymer was purified from the WT strain 10430S as well as the WTA-deficient strain 10403S Δ*tagO1-2*_sup_. Part of the peptidoglycan purification process involves treatment of the sample with concentrated hydrofluoric acid (HF) to remove WTA. However, as the peptidoglycan was also purified from a WTA-deficient strain, binding assays were initially performed with peptidoglycan samples prior to HF treatment. The recombinant His-MBP and His-MBP-GW_Ami_ proteins were incubated in the absence of peptidoglycan (no PG) or in the presence of peptidoglycan isolated from either the WT or WTA-deficient strains and used at concentrations of 2.5, 0.5, or 0.25 mg/ml. Similar to whole cells, the purified peptidoglycan material plus any bound protein was removed by centrifugation. As expected, the His-MBP control protein was recovered in all samples in the supernatant fraction, showing that the MBP does not bind nonspecifically to purified peptidoglycan (Fig. 9). On the other hand, the His-MBP-GW_Ami_ fusion protein bound to the peptidoglycan purified from the WT strain and was therefore no longer found in the supernatant fraction at the highest peptidoglycan concentration or found at reduced levels in the supernatant fraction at the lower peptidoglycan concentrations (Fig. 9A). The His-MBP-GW_Ami_ protein showed even better binding to the peptidoglycan sample isolated from a WTA-deficient L. monocytogenes strain, and no protein was recovered in the supernatant fraction, even at a peptidoglycan concentration of 0.5 mg/ml (Fig. 9B). It has been estimated that WTA constitutes up to 70% of the cell wall dry weight in Listeria (20): hence the increased binding of the His-MBP-GW_Ami_ to the peptidoglycan isolated from the WTA-negative strain compared to the peptidoglycan isolated from a WT strain might be due to the fact that the samples were normalized based on dry weight. Lastly, the peptidoglycan isolated from the WT L. monocytogenes strain treated with concentrated HF to chemically remove WTA was also used in binding assays. The His-MBP-GW_Ami_ protein also bound to this peptidoglycan sample (Fig. 9C), altogether suggesting that the peptidoglycan polymer is sufficient for the retention of GW-domain-containing proteins within the cell wall of L. monocytogenes.

## DISCUSSION

Very little is known about the mechanism by which type I LTA is glycosylated, and to the best of our knowledge, we have identified in the present study the first genetic determinant required for this process, namely, the L. monocytogenes gene *lmo0933* (renamed *gtlA* for glycosyltransferase LTA A) (Fig. 2 and 3). The biological function of the sugar modifications on LTA is currently not known, and no difference in growth rates was observed between a WT L. monocytogenes strain and the *gtlA* mutant under standard laboratory growth conditions (Fig. 4). Based on a comprehensive transcriptomics study on the L. monocytogenes strain EGD-e by Toledo-Arana et al. (49), *gtlA* is predicted to be the first gene of a two-gene operon formed together with *lmo0932* (*lmrg_02031*) coding for a membrane protein of unknown function and predicted to contain a SNARE membrane fusion domain. In the same study, no change in *gtlA* expression was observed during the stationary phase, hypoxia, or at low temperature or during growth in a mouse model of infection compared to growth at 37°C in BHI medium. Only when bacteria were grown in human blood, was a >2-fold decrease in expression observed (49). This indicates that *gtlA* may not be part of the general stress response pathway in L. monocytogenes. A BLAST and synteny analysis suggests that GtlA is present in a number of the other sequenced L. monocytogenes strains (13 strains out of 22 analyzed), as well as in Listeria ivanovii, and Listeria seeligeri; however, it is absent in Listeria grayi, Listeria innocua, or Listeria welshimeri (see Table S4 in the supplemental material). This indicates that in the latter strains, LTA is either not glycosylated or is modified with a different sugar or that an enzyme at a different chromosomal location performs this function.

To investigate a potential biological function of the glycosyl modification on LTA, we assessed its contribution in the retention of noncovalently attached GW-domain-containing proteins within the cell wall of L. monocytogenes. The identification of the genetic determinants required for LTA production and its modifications allowed us to construct L. monocytogenes strains lacking d-alanine or galactose modifications or the complete LTA polymer. Using these strains, we could subsequently assess the contribution of LTA and its modification in the retention of GW-domain-containing proteins in living cells. Somewhat surprisingly, our analysis revealed that neither the modifications on LTA nor the actual LTA polymer was absolutely required for the cell wall retention of the GW-domain-containing proteins (Fig. 5 and 6). Through further work using a WTA-negative strain, as well as purified peptidoglycan, we identified the peptidoglycan polymer as a second binding receptor for GW domain proteins (Fig. 8 and 9). This situation is reminiscent of the binding mode of the major autolysin Atl to the cell wall of Staphylococcus aureus. Atl is a bifunctional enzyme with both amidase and glucosaminidase domains, which are fused to the cell wall binding repeat domains. After multiple processing steps, the amidase domain remains linked to the repeat domains R1a/R1b and R2a/R2b and the glucosaminidase domain is preceded by the repeat domains R3a/R3b. These repeat domains share similarity to the GW domains found in L. monocytogenes proteins and are also, in the case of the Atl protein, essential for binding to the cell wall of S. aureus (50). The repeat domains in Atl also contain the highly conserved glycine-tryptophan (GW) motif, and based on their structural similarity to the eukaryotic Src homology 3 (SH3) domains, they have been grouped along with the GW domains found in L. monocytogenes proteins as SH3b (bacterial SH3) domains (14, 51, 52). Initial experiments indicated a direct interaction between the Atl repeat domains and the peptidoglycan polymer in S. aureus (53), and subsequent studies identified an additional interaction between the repeat domains and the LTA polymer (51, 52). Based on the findings presented in these previous studies and the data obtained in the present study, we suggest that this dual receptor binding mode is also observed in Listeria for GW-domain-containing cell wall binding proteins.

A model for the LTA glycosylation process involving two distinct glycosyltransferases and a sugar-linked undecaprenol phosphate intermediate (sugar-P-C_55_) has been proposed based on biochemical studies performed in the 1980s (30–32) (Fig. 1A). This model is based on the detection of sugar-P-C_55_ intermediates in Streptococcus sanguinis and diverse Bacillus strains, including Bacillus coagulans and Bacillus subtilis. These lipid-linked intermediates are produced in the presence of the respective nucleotide-activated sugars (UDP-sugars), monophosphoryl undecaprenol and membrane protein fractions, indicating that the enzyme catalyzing this step is a membrane-anchored glycosyltransferase (step 1 in Fig. 1A). In order to utilize the nucleotide-activated sugar, the catalytic domain needs to be located in the cytoplasm of the cell. As the LTA backbone is polymerized on the outside the cell, the sugar can therefore only be linked to the polymer on the outside of the cell. Thus, this requires that the sugar-P-C_55_ intermediate is transported across the membrane, likely with the aid of a dedicated transporter protein (step 2 in Fig. 1A). It is then utilized on the outside of the cell by a second membrane-bound glycosyltransferase with an extracellular catalytic domain (step 3 in Fig. 1A), which transfers the sugar onto the final LTA acceptor molecule. Experimental evidence supporting the notion that two distinct glycosyltransferases are involved in this process came from early work performed by Yokoyama et al. showing that the formation of the Gal-P-prenol intermediate in B. coagulans proceeds at an optimal pH of 8.4 and in the presence of MgCl_2_, whereas the transfer of the galactose from this lipid intermediate to the LTA polymer is most efficient at pH 4.5 in the presence of CaCl_2_ (32).

We noted that the proposed LTA glycosylation process has similarities to the mechanism by which 4-amino-4-deoxyl-l-arabinose (l-Ara4N) is linked to lipid A in E. coli and Salmonella enterica serovar Typhimurium (54) and used this information in the present study to identify GtlA (Lmo0933) as the glycosyltransferase involved in the LTA glycosylation process in L. monocytogenes. GtlA has homology to the E. coli glycosyltransferase ArnC, which transfers 4-amino-4-deoxyl-4-formamido-l-arabinose (l-Ara4FN) from UDP-β-l-Ara4FN onto the C_55_-P (34). Nearly 100 different families of glycosyltransferases have now been defined (55); however, the structural fold of glycosyltransferases is limited and the majority of enzymes assume either a GT-A or GT-B fold (55). Recently glycosyltransferases with a third, so called “GT-C” fold, have been defined; this fold is found in integral multi-transmembrane proteins with glycosyltransferase activity (55, 56). The L. monocytogenes GtlA and E. coli ArnC proteins are both GT2 family enzymes with a GT-A fold glycosyltransferase domain at the N-terminal end and are retained within the membrane by two C-terminal transmembrane helices. Based on the analogy to the function of ArnC in the lipid A glycosylation process, we therefore propose that GtlA is the glycosyltransferase required for the production of the Gal-P-C_55_ lipid intermediate, which is subsequently used in the LTA glycosylation process (Fig. 1A, step 1). However, further experiments and a biochemical characterization of the L. monocytogenes GtlA enzyme are required to experimentally confirm this notion.

While in this study we identified the first protein required for the LTA glycosylation process, it is clear that other proteins are required for this process, likely including a transporter protein to move the sugar-P-C_55_ intermediate from the inside to the outside of the membrane and a second glycosyltransferase with extracellular activity. This second glycosyltransferase is likely to be a glycosyltransferase with a GT-C fold, as several enzymes with this fold have been shown to utilize lipid-linked sugars as the substrates (35). However, none of the 15 glycosyltransferases annotated in the Carbohydrate-Active enZYmes (CAZy) database for L. monocytogenes strains EGD-e and 10403S falls into one of the currently known families of glycosyltransferases with a GT-C fold. The homology between known GT-C fold glycosyltransferases is generally very low, and therefore these enzymes are difficult to predict bioinformatically. But this makes it also very likely that additional new families of glycosyltransferases will be uncovered in the future in order to account for all of the cellular processes requiring this class of enzymes.

## Supplementary Material

Supplemental material

## References

[1] Lomonaco S, Nucera D, Filipello V 2015 The evolution and epidemiology of Listeria monocytogenes in Europe and the United States. Infect Genet Evol 35:172–183. doi:10.1016/j.meegid.2015.08.008. 26254574

[2] Cossart P, Lebreton A 2014 A trip in the “New Microbiology” with the bacterial pathogen Listeria monocytogenes. FEBS Lett 588:2437–2445. doi:10.1016/j.febslet.2014.05.051. 24911203

[3] Pizarro-Cerda J, Kuhbacher A, Cossart P 2012 Entry of Listeria monocytogenes in mammalian epithelial cells: an updated view. Cold Spring Harb Perspect Med 2:a010009. doi:10.1101/cshperspect.a010009. 23125201 PMC3543101

[4] Stavru F, Archambaud C, Cossart P 2011 Cell biology and immunology of Listeria monocytogenes infections: novel insights. Immunol Rev 240:160–184. doi:10.1111/j.1600-065X.2010.00993.x. 21349093

[5] Dussurget O, Pizarro-Cerda J, Cossart P 2004 Molecular determinants of Listeria monocytogenes virulence. Annu Rev Microbiol 58:587–610. doi:10.1146/annurev.micro.57.030502.090934. 15487949

[6] Carvalho F, Sousa S, Cabanes D 2014 How Listeria monocytogenes organizes its surface for virulence. Front Cell Infect Microbiol 4:48. doi:10.3389/fcimb.2014.00048. 24809022 PMC4010754

[7] Bierne H, Cossart P 2007 Listeria monocytogenes surface proteins: from genome predictions to function. Microbiol Mol Biol Rev 71:377–397. doi:10.1128/MMBR.00039-06. 17554049 PMC1899877

[8] Dramsi S, Biswas I, Maguin E, Braun L, Mastroeni P, Cossart P 1995 Entry of Listeria monocytogenes into hepatocytes requires expression of *inIB*, a surface protein of the internalin multigene family. Mol Microbiol 16:251–261. doi:10.1111/j.1365-2958.1995.tb02297.x. 7565087

[9] Lingnau A, Domann E, Hudel M, Bock M, Nichterlein T, Wehland J, Chakraborty T 1995 Expression of the Listeria monocytogenes EGD *inlA* and *inlB* genes, whose products mediate bacterial entry into tissue culture cell lines, by PrfA-dependent and -independent mechanisms. Infect Immun 63:3896–3903. 7558297 10.1128/iai.63.10.3896-3903.1995 PMC173548

[10] Parida SK, Domann E, Rohde M, Muller S, Darji A, Hain T, Wehland J, Chakraborty T 1998 Internalin B is essential for adhesion and mediates the invasion of Listeria monocytogenes into human endothelial cells. Mol Microbiol 28:81–93. 9593298 10.1046/j.1365-2958.1998.00776.x

[11] Braun L, Dramsi S, Dehoux P, Bierne H, Lindahl G, Cossart P 1997 InlB: an invasion protein of Listeria monocytogenes with a novel type of surface association. Mol Microbiol 25:285–294. doi:10.1046/j.1365-2958.1997.4621825.x. 9282740

[12] Jonquières R, Bierne H, Fiedler F, Gounon P, Cossart P 1999 Interaction between the protein InlB of Listeria monocytogenes and lipoteichoic acid: a novel mechanism of protein association at the surface of Gram-positive bacteria. Mol Microbiol 34:902–914. doi:10.1046/j.1365-2958.1999.01652.x. 10594817

[13] Banerjee M, Copp J, Vuga D, Marino M, Chapman T, van der Geer P, Ghosh P 2004 GW domains of the Listeria monocytogenes invasion protein InlB are required for potentiation of Met activation. Mol Microbiol 52:257–271. doi:10.1111/j.1365-2958.2003.03968.x. 15049825

[14] Marino M, Banerjee M, Jonquieres R, Cossart P, Ghosh P 2002 GW domains of the Listeria monocytogenes invasion protein InlB are SH3-like and mediate binding to host ligands. EMBO J 21:5623–5634. doi:10.1093/emboj/cdf558. 12411480 PMC131055

[15] Percy MG, Gründling A 2014 Lipoteichoic acid synthesis and function in Gram-positive bacteria. Annu Rev Microbiol 68:81–100. doi:10.1146/annurev-micro-091213-112949. 24819367

[16] Brown S, Santa Maria JP Jr, Walker S 2013 Wall teichoic acids of Gram-positive bacteria. Annu Rev Microbiol 67:313–336. doi:10.1146/annurev-micro-092412-155620. 24024634 PMC3883102

[17] Fiedler F, Seger J, Schrettenbrunner A, Seeliger HPR 1984 The biochemistry of murein and cell-wall teichoic acids in the genus Listeria. Syst Appl Microbiol 5:360–376. doi:10.1016/S0723-2020(84)80038-7.

[18] Fujii H, Kamisango K, Nagaoka M, Uchikawa K, Sekikawa I, Yamamoto K, Azuma I 1985 Structural study on teichoic acids of Listeria monocytogenes types 4a and 4d. J Biochem 97:883–891. 3926758 10.1093/oxfordjournals.jbchem.a135130

[19] Uchikawa K, Sekikawa I, Azuma I 1986 Structural studies on teichoic acids in cell walls of several serotypes of Listeria monocytogenes. J Biochem 99:315–327. 3084460 10.1093/oxfordjournals.jbchem.a135486

[20] Fiedler F 1988 Biochemistry of the cell surface of Listeria strains: a locating general view. Infection 16(Suppl 2):S92–S97. doi:10.1007/BF01639729. 3417357

[21] Eugster MR, Loessner MJ 2012 Wall teichoic acids restrict access of bacteriophage endolysin Ply118, Ply511, and PlyP40 cell wall binding domains to the Listeria monocytogenes peptidoglycan. J Bacteriol 194:6498–6506. doi:10.1128/JB.00808-12. 23002226 PMC3497465

[22] Hether NW, Jackson LL 1983 Lipoteichoic acid from Listeria monocytogenes. J Bacteriol 156:809–817. 6415040 10.1128/jb.156.2.809-817.1983 PMC217899

[23] Uchikawa K, Sekikawa I, Azuma I 1986 Structural studies on lipoteichoic acids from four Listeria strains. J Bacteriol 168:115–122. 3093460 10.1128/jb.168.1.115-122.1986 PMC213427

[24] Reichmann NT, Gründling A 2011 Location, synthesis and function of glycolipids and polyglycerolphosphate lipoteichoic acid in Gram-positive bacteria of the phylum Firmicutes. FEMS Microbiol Lett 319:97–105. doi:10.1111/j.1574-6968.2011.02260.x. 21388439 PMC3089915

[25] Webb AJ, Karatsa-Dodgson M, Gründling A 2009 Two-enzyme systems for glycolipid and polyglycerolphosphate lipoteichoic acid synthesis in Listeria monocytogenes. Mol Microbiol 74:299–314. doi:10.1111/j.1365-2958.2009.06829.x. 19682249 PMC2764115

[26] Gründling A, Schneewind O 2007 Synthesis of glycerol phosphate lipoteichoic acid in Staphylococcus aureus. Proc Natl Acad Sci U S A 104:8478–8483. doi:10.1073/pnas.0701821104. 17483484 PMC1895975

[27] Karatsa-Dodgson M, Wörmann ME, Gründling A 2010 In vitro analysis of the Staphylococcus aureus lipoteichoic acid synthase enzyme using fluorescently labeled lipids. J Bacteriol 192:5341–5349. doi:10.1128/JB.00453-10. 20709894 PMC2950504

[28] Campeotto I, Percy MG, MacDonald JT, Forster A, Freemont PS, Gründling A 2014 Structural and mechanistic insight into the Listeria monocytogenes two-enzyme lipoteichoic acid synthesis system. J Biol Chem 289:28054–28069. doi:10.1074/jbc.M114.590570. 25128528 PMC4192460

[29] Abachin E, Poyart C, Pellegrini E, Milohanic E, Fiedler F, Berche P, Trieu-Cuot P 2002 Formation of d-alanyl-lipoteichoic acid is required for adhesion and virulence of Listeria monocytogenes. Mol Microbiol 43:1–14. doi:10.1046/j.1365-2958.2002.02723.x. 11849532

[30] Iwasaki H, Shimada A, Yokoyama K, Ito E 1989 Structure and glycosylation of lipoteichoic acids in Bacillus strains. J Bacteriol 171:424–429. 2914853 10.1128/jb.171.1.424-429.1989 PMC209605

[31] Mancuso DJ, Chiu TH 1982 Biosynthesis of glucosyl monophosphoryl undecaprenol and its role in lipoteichoic acid biosynthesis. J Bacteriol 152:616–625. 7130126 10.1128/jb.152.2.616-625.1982 PMC221508

[32] Yokoyama K, Araki Y, Ito E 1988 The function of galactosyl phosphorylpolyprenol in biosynthesis of lipoteichoic acid in Bacillus coagulans. Eur J Biochem 173:453–458. doi:10.1111/j.1432-1033.1988.tb14020.x. 3360021

[33] Fischer W 1994 Lipoteichoic acid and lipids in the membrane of Staphylococcus aureus. Med Microbiol Immunol 183:61–76. doi:10.1007/BF00277157. 7935161

[34] Breazeale SD, Ribeiro AA, McClerren AL, Raetz CR 2005 A formyltransferase required for polymyxin resistance in Escherichia coli and the modification of lipid A with 4-amino-4-deoxy-l-arabinose. Identification and function oF UDP-4-deoxy-4-formamido-l-arabinose. J Biol Chem 280:14154–14167. 15695810 10.1074/jbc.M414265200

[35] Alderwick LJ, Lloyd GS, Ghadbane H, May JW, Bhatt A, Eggeling L, Futterer K, Besra GS 2011 The C-terminal domain of the arabinosyltransferase Mycobacterium tuberculosis EmbC is a lectin-like carbohydrate binding module. PLoS Pathog 7:e1001299. doi:10.1371/journal.ppat.1001299. 21383969 PMC3044687

[36] Berg S, Kaur D, Jackson M, Brennan PJ 2007 The glycosyltransferases of Mycobacterium tuberculosis—roles in the synthesis of arabinogalactan, lipoarabinomannan, and other glycoconjugates. Glycobiology 17:35–56R. 17261566 10.1093/glycob/cwm010

[37] Horton RM, Hunt HD, Ho SN, Pullen JK, Pease LR 1989 Engineering hybrid genes without the use of restriction enzymes: gene splicing by overlap extension. Gene 77:61–68. doi:10.1016/0378-1119(89)90359-4. 2744488

[38] Camilli A, Tilney LG, Portnoy DA 1993 Dual roles of plcA in Listeria monocytogenes pathogenesis. Mol Microbiol 8:143–157. doi:10.1111/j.1365-2958.1993.tb01211.x. 8388529 PMC4836944

[39] Morath S, Geyer A, Hartung T 2001 Structure-function relationship of cytokine induction by lipoteichoic acid from Staphylococcus aureus. J Exp Med 193:393–397. doi:10.1084/jem.193.3.393. 11157059 PMC2195914

[40] Morath S, Geyer A, Spreitzer I, Hermann C, Hartung T 2002 Structural decomposition and heterogeneity of commercial lipoteichoic acid preparations. Infect Immun 70:938–944. doi:10.1128/IAI.70.2.938-944.2002. 11796629 PMC127707

[41] Morath S, Stadelmaier A, Geyer A, Schmidt RR, Hartung T 2002 Synthetic lipoteichoic acid from Staphylococcus aureus is a potent stimulus of cytokine release. J Exp Med 195:1635–1640. doi:10.1084/jem.20020322. 12070290 PMC2193559

[42] Wolters PJ, Hildebrandt KM, Dickie JP, Anderson JS 1990 Polymer length of teichuronic acid released from cell walls of Micrococcus luteus. J Bacteriol 172:5154–5159. 2394683 10.1128/jb.172.9.5154-5159.1990 PMC213175

[43] Lu D, Wörmann ME, Zhang X, Schneewind O, Gründling A, Freemont PS 2009 Structure-based mechanism of lipoteichoic acid synthesis by Staphylococcus aureus LtaS. Proc Natl Acad Sci U S A 106:1584–1589. doi:10.1073/pnas.0809020106. 19168632 PMC2635763

[44] de Jonge BL, Chang YS, Gage D, Tomasz A 1992 Peptidoglycan composition of a highly methicillin-resistant Staphylococcus aureus strain. The role of penicillin binding protein 2A. J Biol Chem 267:11248–11254. 1597460

[45] Oberto J 2013 SyntTax: a web server linking synteny to prokaryotic taxonomy. BMC Bioinformatics 14:4. doi:10.1186/1471-2105-14-4. 23323735 PMC3571937

[46] Eugster MR, Haug MC, Huwiler SG, Loessner MJ 2011 The cell wall binding domain of Listeria bacteriophage endolysin PlyP35 recognizes terminal GlcNAc residues in cell wall teichoic acid. Mol Microbiol 81:1419–1432. doi:10.1111/j.1365-2958.2011.07774.x. 21790805

[47] Becavin C, Bouchier C, Lechat P, Archambaud C, Creno S, Gouin E, Wu Z, Kuhbacher A, Brisse S, Pucciarelli MG, Garcia-del Portillo F, Hain T, Portnoy DA, Chakraborty T, Lecuit M, Pizarro-Cerda J, Moszer I, Bierne H, Cossart P 2014 Comparison of widely used Listeria monocytogenes strains EGD, 10403S, and EGD-e highlights genomic variations underlying differences in pathogenicity. mBio 5:e00969-14. doi:10.1128/mBio.00969-14. 24667708 PMC3977354

[48] Lu YJ, Zhang YM, Grimes KD, Qi J, Lee RE, Rock CO 2006 Acyl-phosphates initiate membrane phospholipid synthesis in Gram-positive pathogens. Mol Cell 23:765–772. doi:10.1016/j.molcel.2006.06.030. 16949372

[49] Toledo-Arana A, Dussurget O, Nikitas G, Sesto N, Guet-Revillet H, Balestrino D, Loh E, Gripenland J, Tiensuu T, Vaitkevicius K, Barthelemy M, Vergassola M, Nahori MA, Soubigou G, Regnault B, Coppee JY, Lecuit M, Johansson J, Cossart P 2009 The Listeria transcriptional landscape from saprophytism to virulence. Nature 459:950–956. doi:10.1038/nature08080. 19448609

[50] Baba T, Schneewind O 1998 Targeting of muralytic enzymes to the cell division site of Gram-positive bacteria: repeat domains direct autolysin to the equatorial surface ring of Staphylococcus aureus. EMBO J 17:4639–4646. doi:10.1093/emboj/17.16.4639. 9707423 PMC1170793

[51] Götz F, Heilmann C, Stehle T 2014 Functional and structural analysis of the major amidase (Atl) in Staphylococcus. Int J Med Microbiol 304:156–163. doi:10.1016/j.ijmm.2013.11.006. 24444718

[52] Zoll S, Schlag M, Shkumatov AV, Rautenberg M, Svergun DI, Götz F, Stehle T 2012 Ligand-binding properties and conformational dynamics of autolysin repeat domains in staphylococcal cell wall recognition. J Bacteriol 194:3789–3802. doi:10.1128/JB.00331-12. 22609916 PMC3416534

[53] Biswas R, Voggu L, Simon UK, Hentschel P, Thumm G, Götz F 2006 Activity of the major staphylococcal autolysin Atl. FEMS Microbiol Lett 259:260–268. doi:10.1111/j.1574-6968.2006.00281.x. 16734789

[54] Yan A, Guan Z, Raetz CR 2007 An undecaprenyl phosphate-aminoarabinose flippase required for polymyxin resistance in Escherichia coli. J Biol Chem 282:36077–36089. doi:10.1074/jbc.M706172200. 17928292 PMC2613183

[55] Lairson LL, Henrissat B, Davies GJ, Withers SG 2008 Glycosyltransferases: structures, functions, and mechanisms. Annu Rev Biochem 77:521–555. doi:10.1146/annurev.biochem.76.061005.092322. 18518825

[56] Liu J, Mushegian A 2003 Three monophyletic superfamilies account for the majority of the known glycosyltransferases. Protein Sci 12:1418–1431. doi:10.1110/ps.0302103. 12824488 PMC2323934

