# Supplementary material for: Identification of a Lipoteichoic Acid Glycosyltransferase Enzyme Reveals that GW-Domain-Containing Proteins Can Be Retained in the Cell Wall of Listeria monocytogenes in the Absence of Lipoteichoic Acid or Its Modifications
Source: J Bacteriol. 2016 Jul 13;198(15):2029–42. doi: 10.1128/JB.00116-16 (PMC4944223; doi:10.1128/JB.00116-16)
Supplement: Supplemental material [file supp_198_15_2029__index.html]

Supplemental material 

# Identification of a Lipoteichoic Acid Glycosyltransferase Enzyme Reveals that GW-Domain-Containing Proteins Can Be Retained in the Cell Wall of Listeria monocytogenes in the Absence of Lipoteichoic Acid or Its Modifications

## Supplemental material

- Supplemental file 1 -

  Tables S1 (Strains), S2 (Primers), S3 (Genome sequence comparison), and S4 (Distribution of GtlA and Lmo2500 homologs in different *Listeria* species)

  PDF, 202K
