# Supplementary material for: Identification of a Lipoteichoic Acid Glycosyltransferase Enzyme Reveals that GW-Domain-Containing Proteins Can Be Retained in the Cell Wall of Listeria monocytogenes in the Absence of Lipoteichoic Acid or Its Modifications
Source: J Bacteriol. 2016 Jul 13;198(15):2029–42. doi: 10.1128/JB.00116-16 (PMC4944223; doi:10.1128/JB.00116-16)
Supplement: Supplemental material [file JB.00116-16_zjb999094114so1.pdf]

Supplemental Material

TABLES

TABLE S1 Bacterial strains used in this study

Strain	Relevant features	Reference
	***Escherichia coli* strains**	
XL1-Blue	Cloning strain; TetR – ANG127	Stratagene
SM10	*E. coli* strain used for conjugations; KanR – ANG618	(1)
BL21(DE3)	Protein expression strain –ANG191	Novagen
DH-E898	XL1 Blue pPL3; *L. monocytogenes* integration vector; CamR – ANG1276	(2)
DH-E899	XL1 Blue pHPL3; *L. monocytogenes* integration vector with hyper-spac promoter; CamR – ANG1277	(2)
DH-E964	XL1 Blue pPL3e; *L. monocytogenes* integration vector; ErmR – ANG1278	(2)
ANG103	XL1-Blue pQE30-GFP; AmpR	(3)
ANG1264	DH5α pKSV7; allelic exchange vector; AmpR	(4)
ANG1375	SM10 pHPL3; *E. coli* conjugation strain with plasmid pHPL3; KanR, CamR	This study
ANG1378	CLG190 pKSV7Δ*lmo0644* (Δ*ltaP*); AmpR	(5)
ANG1380	XL1-Blue pKSV7Δ*tagO1* (Δ*lmo0959*); AmpR	This study
ANG1381	XL1-Blue pKSV7Δ*tagO2* (Δ*lmo2519*); AmpR	This study
ANG1394	XL1-Blue pPL3-*tagO1*; CamR	This study
ANG1395	XL1-Blue pHPL3-*tagO2*; CamR	This study
ANG1456	SM10 pPL3; *E. coli* conjugation strain with plasmid pPL3; KanR, CamR	(5)
ANG1551	XL1-Blue pHPL3-*inlB-GW-His*; CamR	This study
ANG1625	SM10 pHPL3-*inlB-GW-His*; KanR, CamR	This study
ANG1646	XL1-Blue pKSV7Δ*dltA* (Δ*lmo0974*); AmpR	This study
ANG1807	SM10 pPL3-*tagO1*; *E. coli* conjugation strain with plasmid pPL3-*tagO1*; KanR, CamR	This study
ANG1808	SM10 pHPL3-*tagO2*; *E. coli* conjugation strain with plasmid pHPL3-*tagO2*; KanR, CamR	This study
ANG1975	XL1-Blue pHPL3-*inlB-*GW_{Ami}*-His*; CamR	This study
ANG2014	SM10 pHPL3-*inlB-*GW_{Ami}*-His*; KanR, CamR	This study
ANG2157	XL1-Blue pQE30-GFP-GW_{Ami}; AmpR	This study
ANG2222	XL1-Blue pKSV7Δ*gtlA* (Δ*lmo0933*); AmpR	This study
ANG2322	XL1-Blue pPL3e-*gtlA*; CamR	This study
ANG2816	XL1-Blue pVL847; AmpR	(6)
ANG2890	BL21(DE3) pVL847; AmpR	(7)
ANG3179	XL1-Blue pVL847-GW_{Ami}; AmpR	This study
ANG3181	BL21(DE3) pVL847-GW_{Ami}; AmpR	This study
	***Listeria monocytogenes* strains**	
10403S	StrepR – ANG1263	(8)
EGD	*L. monocytgenes* strain with *prfA** mutation - ANG4004	(9)
ANG1386	10403SΔ*ltaS* (Δ*lmo0927*); StrepR	(5)
ANG1387	10403SΔ*tagO1*; StrepR	This study
ANG1388	10403SΔ*tagO2*; StrepR	This study
ANG1414	10403S pHPL3; StrepR, CamR	(5)

ANG1626	10403S pHPL3-*inlB-GW-His*; StrepR, CamR	This study
ANG1630	10403SΔ*ltaS*$_{sup}$ pHPL3-*inlB-GW-His*; StrepR, CamR	This study
ANG1783	10403SΔ*dltA*; StrepR	This study
ANG1784	10403SΔ*tagO1-2;* StrepR	This study
ANG1832	10403SΔ*tagO1-2* pPL3-*tagO1*; StrepR, CamR	This study
ANG1833	10403SΔ*tagO1-2* pHPL3-*tagO2*; StrepR, CamR	This study
ANG1911	10403SΔ*tagO1-2* pHPL3; StrepR, CamR	This study
ANG2037	10403SΔ*ltaS*$_{sup}$ pHPL3-*inlB-GW*$_{Ami}$*-His*; StrepR, CamR	This study
ANG2015	10403S pHPL3-*inlB-GW*$_{Ami}$*-His*; StrepR, CamR	This study
ANG2325	10403SΔ*gtlA*; StrepR	This study
ANG2337	10403SΔ*ltaS*$_{sup}$; StrepR	This study
ANG2350	10403SΔ*tagO1-2*$_{sup}$; StrepR	This study
ANG2495	10403SΔ*gtlA* pPL3e-*gtlA*; StrepR, ErmR	This study
ANG2496	10403SΔ*gtlA* pPL3e; StrepR, ErmR	This study
ANG2498	10403S pPL3e; StrepR, ErmR	This study
ANG2513	10403SΔ*tagO1-2*$_{sup}$ pHPL3-*inlB-GW-His*; StrepR, CamR	This study
ANG2514	10403SΔ*tagO1-2*$_{sup}$ pHPL3-*inlB-GW*$_{Ami}$*-His*; StrepR, CamR	This study
ANG3325	10403SΔ*ltaP*Δ*ltaS*$_{sup}$ pHPL3-*inlB-GW-His*; StrepR, CamR	This study
ANG3326	10403SΔ*ltaP*Δ*ltaS*$_{sup}$ pHPL3-*inlB-GW*$_{Ami}$*-His*; StrepR, CamR	This study
ANG3330	10403SΔ*gtlA* pHPL3-*inlB-GW-His*; StrepR, CamR	This study
ANG3331	10403SΔ*gtlA* pHPL3-*inlB-GW*$_{Ami}$*-His*; StrepR, CamR	This study
ANG3333	10403SΔ*dltA* pHPL3-*inlB-GW-His*; StrepR, CamR	This study
ANG3334	10403SΔ*dltA* pHPL3-*inlB-GW*$_{Ami}$*-His*; StrepR, CamR	This study
ANG3465	10403SΔ*ltaP*Δ*ltaS*$_{sup}$; StrepR	This study

Antibiotics were used at the following concentrations: for *E. coli* cultures: Kanamycin (KanR) 30 µg/ml, Ampicillin (AmpR) 100 µg/ml, Tetracycline (TetR) 10 µg/ml; Chloramphenicol (CamR) 20 µg/ml; for *L. monocytogenes* cultures: Chloramphenicol (CamR) 7.5 or 10 µg/ml; Erythromycin (ErmR) 5 µg/ml; Streptomycin (StrepR) 200 µg/ml for conjugation experiment.

TABLE S2 Primers used in this study

Number	Name	Sequence
ANG530	5-KpnI-*lmo0959*	GGGGTACCCATCCAGATGTTACTGTGATTCTTGACAACG
ANG531	5-int-*lmo0959*	CTTTTGTTAATTTCGCGCCAAAACATATAAATATAATCATCCAG
ANG532	3-int-*lmo0959*	TGTTTTGGCGCGAAATTAACAAAAGAAGAAAAAATAGATTAGAG
ANG533	3-BamHI-*lmo0959*	CGGGATCCGAGTAGCTTCTTTCGCTTCAACTAACAGTGATG
ANG537	5-KpnI-*lmo2519*	GGGGTACCCTTGCGCAGAAAGAAATACGAACTCCTCAAG
ANG538	5-int-*lmo2519*	GTATTTTTTGAAGTGCAAAGCTAATCAATATACTCCATATTAG
ANG539	3-int-*lmo2519*	GATTAGCTTTGCACTTCAAAAAATACATCGAAAACGAAAATAA
ANG540	3-BamHI-*lmo2519*	CGGGATCCCGGAAAAGCCTCTACACTTCGAACGAATG
ANG655	5-BamHI-*lmo0959*-pPL3	CGGGATCCGCTAGGATTAAACAATAAATAAAAGC
ANG657	5-BamHI-*lmo2519*-pHPL3	CGGGATCCTTTTGCTAAATAAAGGATGGAGCTG
ANG658	3-KpnI-*lmo2519*-pHPL3	GGGGTACCTTATTTTCGTTTTCGATGTATTTTTTGAAGTA
ANG679	3-KpnI-*lmo0959*-pPL3	GGGGTACCGTTTTTCTCCTCATTTCTACAAAAACGTTAC
ANG835	5-BamHI-InlB_pHPL3	CGGGATCCCTAGAATCAAGGAGAGGATAGT
ANG837	3-SalI-InlB-C-His6	ACGCGTCGACTTAGTGATGGTGATGGTGATGACCTTTCTGTGCCCTTAAATTAGCTG CTTTCGTC
ANG932	5-KpnI-dltA	GGGGTACCCCAAAATATAGTTCCCGTTATTTGTCAC
ANG933	5-int-dltA	CATAGTTAACGGATCAATTCTTTCTATGATACTCGTTGTCAT
ANG934	3-int-dltA	GAAAGAATTGATCCGTTAACTATGAATGGCAAAATTGATCGC
ANG935	3-BamHI-dltA	CGGGATCCGAAACGCATGAATACATAATCGCGGAACC
ANG1119	3-SalI-Ami-C-His6	ACGCGTCGACTTAGTGATGGTGATGGTGATGACCTTGCTTTTTAGCACTTAGGTTAG CAGCTTT
ANG1172	3-InlB_Ami-fusion	TTTTTCGTTAATGTTGACTGTTTTTTCGGTCGTTTC
ANG1173	5-InlB_Ami-fusion	AAAACAGTCAACATTAACGAAAAATATAAAGCAATG
ANG1298	5-BamHI-1kb-lmo0933	CGGGATCCGCTCAATTAAAAAAGTTTCGAGTAGC
ANG1299	front-int-lmo0933	ACCATTTTCTTCTATACAGGAGTATTCAATACTATTTTCCAT
ANG1300	back-int-lmo0933	TACTCCTGTATAGAAGAAAATGGTTTTTCTGACCAAAAATAA
ANG1301	3-KpnI-1kb-lmo0933	GGGGTACCGGTGACTTCTTCGGCGTTAAAAATGTGG
ANG1320	5-SacI-Ami-AA263-GWs	ATCAGAGCTCATTAACGAAAAATATAAAGCAATGCAAG
ANG1321	3-SalI-Ami-AA263-GWs	ACGCGTCGACTTATTGCTTTTTAGCACTTAGGTTAGCAGC
ANG1421	5-BamHI-Lmo0933 with P	GGGGATCCCATCCCCTCTCTCCTTATTTTCAGGC
ANG1422	3-KpnI-Lmo0933 end	CGGGTACCTTATTTTTGGTCAGAAAAACCATTTTC
ANG1806	5-NdeI-Ami-GW(263)	TAATAACCATATGATTAACGAAAAATATAAAGCAATGCAAG
ANG1807	3-BamHI-Ami	CGGGATCCTTATTGCTTTTTAGCACTTAGGTTAGCAGC

Restriction sites in primer sequences are underlined

TABLE 3 Genome sequence comparison of strains 10403S, 10403SΔ*ltaS*$_{sup}$ and 10403SΔ*tagO1-2*$_{sup}$ with the published sequence of stain 10403S (NC_017544.1)

**10403S**							
**Reference Position**	**Type**	**Ref.**	**Allele**	**Freq.**	**Av. quality**	**Annotations**	**Amino acid change**
450115	SNV	A	G	100	36.907	LMRG_00121, hypothetical proteins potential - Alpha-mannosidase [Carbohydrate transport and metabolism]	Silent
961124	SNV	C	A	100	36.577	LMRG_02041, Non-specific DNA-binding protein Dps / Iron-binding ferritin-like antioxidant protein/Ferroxidase (EC 1.16.3.1)	Thr90Lys
1443034	SNV	C	T	100	37.409	LMRG_00903, 4-hydroxy-3-methylbut-2-enyl diphosphate reductase; IspH	Pro189Leu
2372343	SNV	G	C	100	35.054	LMRG_01494, L-Cystine ABC transporter, periplasmic cystine-binding protein TcyK	Pro73Arg
**10403SΔ*ltaS*$_{sup}$**							
**Reference Position**	**Type**	**Ref.**	**Allele**	**Freq.**	**Av. quality**	**Annotations**	**Amino acid change**
450115	SNV	A	G	100	35.75	LMRG_00121, hypothetical proteins potential - Alpha-mannosidase [Carbohydrate transport and metabolism]	Silent
961124	SNV	C	A	100	37.727	LMRG_02041, Non-specific DNA-binding protein Dps / Iron-binding ferritin-like antioxidant protein/Ferroxidase (EC 1.16.3.1)	Thr90Lys
1443034	SNV	C	T	100	37.526	LMRG_00903, 4-hydroxy-3-methylbut-2-enyl diphosphate reductase; IspH	Pro189Leu
2372343	SNV	G	C	100	35.136	LMRG_01494, L-Cystine ABC transporter, periplasmic cystine-binding protein TcyK	Pro73Arg
12978	SNV	C	T	100	37.269	LMRG_02439, Mevalonate kinase [Lipid metabolism];	Silent
424538	SNV	A	G	92	37	LMRG_00095,Transcriptional antiterminator; PRD/PTS system IIA 2 domain protein	Tyr362Cys
599542	SNV	A	C	100	37.091	LMRG_00264, Invasion associated secreted endopeptidase Iap (p60)	Silent
1127012	SNV	C	T	100	36.741	intergenic region between LMRG_00575 (hypothetical protein) and LMRG_00576 (MerR family transcriptional regulator)	Intergenic region
1164987	SNV	G	C	100	37	LMRG_00623, ethanolamine utilization protein EutL	Gly37Arg
1907291	SNV	T	C	100	35.5	intergenic region between LMRG_01022 (ATPase components of ABC transporters with duplicated ATPase domains) and LMRG_01023 (Formate--tetrahydrofolate ligase (EC 6.3.4.3)	Intergenic region
2537338	Insertion	-	T	89	37	LMRG_01743, D-glutamyl-L-m-Dpm peptidase P45	Leu85fs
2596901	SNV	T	G	100	36.84	LMRG_02708, Arginyl-tRNA synthetase [Translation,_ribosomal structure and biogenesis];	Glu29Ala
**10403SΔ*tagO1-2*$_{sup}$**							
**Reference Position**	**Type**	**Ref.**	**Allele**	**Freq.**	**Av. quality**	**Annotations**	**Amino acid change**
450115	SNV	A	G	100	36.292	LMRG_00121, hypothetical proteins potential - Alpha-mannosidase [Carbohydrate transport and metabolism]	Silent
961124	SNV	C	A	100	36.414	LMRG_02041, Non-specific DNA-binding protein Dps / Iron-binding ferritin-like antioxidant protein/Ferroxidase (EC 1.16.3.1)	Thr90Lys
1443034	SNV	C	T	100	36.958	LMRG_00903, 4-hydroxy-3-methylbut-2-enyl diphosphate reductase; IspH	Pro189Leu
2372343	SNV	G	C	100	34.647	LMRG_01494, L-Cystine ABC transporter, periplasmic cystine-binding protein TcyK	Pro73Arg
1840272	SNV	C	T	100	35.875	LMRG_00956, acyl-acyl carrier protein phosphate acyltransferase PlsX	Met104Ile

Rows shaded in grey are genomic alterations present in both, WT and mutant strains while rows without shading are mutations specific to the LTA or WTA negative suppressor strains.

TABLE 4 Distribution of GtlA (Lmo0933) and Lmo2500 homologs in different *Listeria* species

TABLE S4 - Distribution of GtlA (Lmo0933) and Lmo2500 homologs in different *Listeria* species

**Strain**	**Lmo0933**	**Lmo2550**
Listeria_ivanovii_subsp_ivanovii_ bp257573	✔	✗
Listeria_ivanovii_subsp_ivanovii_PAM_55_bp73473	✔	✗
Listeria_ivanovii_WSLC3009_bp236792	✔	✗
Listeria_monocytogenes_08_5578_bp43671_C1	✔	✔
Listeria_monocytogenes_08_5923_bp43727	✔	✔
Listeria_monocytogenes_10403S_bp54461	✔	✔
Listeria_monocytogenes_bp193768	✔	✔
Listeria_monocytogenes_EGD_bp223288	✔	✔
Listeria_monocytogenes_EGD_e_bp61583	✔	✔
Listeria_monocytogenes_Finland_1998_bp54443	✔	✔
Listeria_monocytogenes_J0161_bp54459	✔	✔
Listeria_monocytogenes_FSL_R2_561_bp54441	✔	✔
Listeria_monocytogenes_N53_1_bp193767	✔	✔
Listeria_monocytogenes_SLCC2755_bp52455_C1	✔	✔
Listeria_monocytogenes_WSLC1001_bp236721	✔	✔
Listeria_seeligeri_serovar_1_2b_str_SLCC3954_bp46215	✔	✔
Listeria_innocua_Clip11262_bp61567_C1	✗	✔
Listeria_welshimeri_serovar_6b_str_SLCC5334_bp61605	✗	✔
Listeria_monocytogenes_07PF0776_bp162185	✗	✗
Listeria_monocytogenes_HCC23_bp59203	✗	✗
Listeria_monocytogenes_J1816_bp179734	✗	✗
Listeria_monocytogenes_J1_220_bp179735	✗	✗
Listeria_monocytogenes_M7_bp162131	✗	✗
Listeria_monocytogenes_serotype_4b_str_CLIP_80459_bp59317	✗	✗
Listeria_monocytogenes_serotype_4b_str_F2365_bp57689	✗	✗
Listeria_monocytogenes_serotype_4b_str_LL195_bp182103	✗	✗
Listeria_monocytogenes_WSLC1042_bp236791	✗	✗
Listeria_grayi_bp52457	✗	✗
Listeria_ivanovii_subsp_londoniensis_bp257575	✗	✗
Listeria_monocytogenes_FSL_J1_208_bp181531_C1	✗	✗

Checkmark indicates presence and cross sign indicates absence of the specified glycosyltransferase.

REFERENCES

1. **Simon R, Priefer U, Pühler A.** 1983. A Broad Host Range Mobilization System for *In Vivo* Genetic Engineering: Transposon Mutagenesis in Gram-Negative Bacteria. Nature Biotechnology **1:**784-791.
2. **Gründling A, Burrack LS, Bouwer HG, Higgins DE.** 2004. *Listeria monocytogenes* regulates flagellar motility gene expression through MogR, a transcriptional repressor required for virulence. Proc Natl Acad Sci U S A **101:**12318-12323.
3. **Gründling A, Schneewind O.** 2006. Cross-linked peptidoglycan mediates lysostaphin binding to the cell wall envelope of *Staphylococcus aureus*. Journal of bacteriology **188:**2463-2472.
4. **Smith K, Youngman P.** 1992. Use of a new integrational vector to investigate compartment-specific expression of the *Bacillus subtilis spoIIM* gene. Biochimie **74:**705-711.
5. **Webb AJ, Karatsa-Dodgson M, Gründling A.** 2009. Two-enzyme systems for glycolipid and polyglycerolphosphate lipoteichoic acid synthesis in *Listeria monocytogenes*. Mol Microbiol **74:**299-314.
6. **Lee VT, Matewish JM, Kessler JL, Hyodo M, Hayakawa Y, Lory S.** 2007. A cyclic-di-GMP receptor required for bacterial exopolysaccharide production. Mol Microbiol **65:**1474-1484.
7. **Corrigan RM, Bowman L, Willis AR, Kaever V, Gründling A.** 2015. Cross-talk between Two Nucleotide-signaling Pathways in *Staphylococcus aureus*. J Biol Chem **290:**5826-5839.
8. **Bishop DK, Hinrichs DJ.** 1987. Adoptive transfer of immunity to *Listeria monocytogenes*. The influence of in vitro stimulation on lymphocyte subset requirements. J Immunol **139:**2005-2009.
9. **Mackaness GB.** 1962. Cellular resistance to infection. J Exp Med **116:**381-406.
